# Taxonomy and Phylogeny of Fungi Associated with *Mangifera indica* from Yunnan, China

**DOI:** 10.3390/jof8121249

**Published:** 2022-11-26

**Authors:** Er-Fu Yang, Samantha C. Karunarathna, Dong-Qin Dai, Steven L. Stephenson, Abdallah M. Elgorban, Salim Al-Rejaie, Yin-Ru Xiong, Itthayakorn Promputtha, Milan C. Samarakoon, Saowaluck Tibpromma

**Affiliations:** 1 Center for Yunnan Plateau Biological Resources Protection and Utilization, College of Biological Resource and Food Engineering, Qujing Normal University, Qujing 655011, China; 2 Department of Biology, Faculty of Science, Chiang Mai University, Chiang Mai 50200, Thailand; 3 Master of Science Program in Applied Microbiology (International Program), Faculty of Science, Chiang Mai University, Chiang Mai 50200, Thailand; 4 Department of Biological Sciences, University of Arkansas, Fayetteville, AR 72701, USA; 5 Department of Botany and Microbiology, College of Science, King Saud University, Riyadh 12211, Saudi Arabia; 6 Department of Pharmacology & Toxicology, College of Pharmacy, King Saud University, Riyadh 12211, Saudi Arabia; 7 Innovative Institute for Plant Health, Zhongkai University of Agriculture and Engineering, Guangzhou 510225, China; 8 Environmental Science Research Center, Faculty of Science, Chiang Mai University, Chiang Mai 50200, Thailand; 9 Department of Entomology and Plant Pathology, Faculty of Agriculture, Chiang Mai University, Chiang Mai 50200, Thailand

**Keywords:** mango, plant-associated microfungi, saprobic fungi

## Abstract

During investigations of saprobic fungi associated with mango (*Mangifera indica*) in Baoshan and Honghe of Yunnan Province (China), fungal taxa belonging to the orders Botryosphaeriales, Calosphaeriales, Chaetothyriales, Diaporthales, and Xylariales were recorded. Morphological examinations coupled with phylogenetic analyses of multigene sequences (ITS, LSU, SSU, *tef1-α*, *rpb1*, *rpb2,* β-tubulin and CAL) were used to identify the fungal taxa. A new genus viz. *Mangifericola,* four new species viz. *Cyphellophora hongheensis, Diaporthe hongheensis, Hypoxylon hongheensis,* and *Mangifericola hongheensis,* four new host and geographical records viz. *Aplosporella artocarpi*, *Hypomontagnella monticulosa, Paraeutypella citricola* and *Pleurostoma ootheca,* and two new collections of *Lasiodiplodia* are reported.

## 1. Introduction

Mango (*Mangifera indica* L.) is a dicotyledonous fruit plant in the family Anacardiaceae, and the genus *Mangifera* contains approximately 69 species with more than 1000 varieties [1]. Mango cultivation history can be traced back 4000 years in India and Southeast Asia. As one of the five most economically significant fruit crops worldwide, Mango is cultivated in more than 100 countries, of them, Asian countries account for approximately 77% of the world’s mango production [2,3,4]. India is the largest mango producer, accounting for about 54.2% of the total mangoes produced worldwide, while China, Thailand, Indonesia, Mexico, and Pakistan are other major mango producers [2,3,4]. Mango planting is an important part of agricultural exports in sub-tropical to tropical countries [3,4,5]. Naturally, mango trees grow best in lowland subtropical to tropical regions, best in dry, sandy soil with a pH of 5.5–7.5, and direct sun is preferred for tree growth and fruit production [6].

The investigation of plant-associated microfungi is important, as is relevant to the trend towards globalization of agricultural markets, including forest and horticultural products [7]. To date, around 2250 records of mango-associated microfungi have been documented in the U.S. National Fungus Collections Fungal Database (https://nt.ars-grin.gov/fungaldatabases/, accessed on 7 November 2022). However, around 205 records (9%) were in India, while 120 records (5.3%) were reported in China. China is the third main mango producer in the world, but the study of mango-associated microfungi is still poorly understood and many more undiscovered species are still waiting to be investigated [5,8]. In addition, the most studied microfungi associated with mango are pathogens and most species reported belonging to *Alternaria*, *Colletotrichum, Fusarium,* and *Rhinocladium* [8,9,10], while the endophytic fungi associated with mango were poorly studied, and most studies lack the combination of morphology and phylogeny [11,12]. Meanwhile, mango-related saprobic fungi have mostly been overlooked. Recently, Yang et al. [13] described ten species/generic records belonging to Pleosporales that were associated with mango and *Mangifericomes* was described as a unique genus in the Pleosporales *incertae sedis* with detailed morphological characteristics and phylogenetic analyses. Therefore, studies of saprobic fungi associated with mango based on morphology and phylogeny are still needed to understand the fungal diversity of mango.

This is the second of the paper series on the fungi associated with *Mangifera indica* from Yunnan, China. In this paper, we introduce a new genus, two new species, and two new host records in the Xylariales, two new species in each of the orders Chaetothyriales and Diaporthales; one new host and country records in the Calosphaeriales, one new host and country records and two known records in the Botryosphaeriales.

## 2. Materials and Methods

### 2.1. Sampling, Isolation and Cultivation of Fungi

The specimens included in the present study were collected from mango plantations in Baoshan, and unmanaged mango trees in Honghe. The dry branches and twigs of mango with black fungal fruiting bodies were randomly picked from Keitt and Guifei mango varieties. The Global Positioning System (GPS) with the altitude, latitude, and longitude of collection sites were recorded. The collected specimens were put in sterilized plastic bags with collection details and brought back to the mycological laboratory at Kunming Institute of Botany, Chinese Academy of Sciences, and stored at room temperature. The microscope slides with fungal microstructures were prepared by an Olympus SZ61 stereo microscope (Japan), and micro-morphological characteristics were captured by a digital camera (Canon EOS 600D, Canon Inc., Tokyo, Japan) on a compound microscope (Nikon ECLIPSE Ni, Nikon., Tokyo, Japan). Measurements of ascomata, peridium, asci, conidiogenous cells and ascospores/conidia were done by the Tarosoft (R) Image Frame Work program. Adobe Photoshop CS3 Extended v. 10.0 (Adobe^®^, San Jose, CA, USA) was used for making the color photo plates.

The single spore isolation was done according to Senanayake et al. [14], and the specimens were observed under an Olympus SZ61 stereo microscope (Japan). The spores/conidial masses were picked up using a sterilized surgical needle and transferred into sterilized water droplets for spore suspension. The spore suspension was spread onto the surface of potato dextrose agar (PDA), and incubated at 27 °C overnight. Later, germinated spores were transferred to PDA and incubated at 27 °C for long-term observation. Culture characteristics such as colony texture, diameter, pigments, and growth rate were recorded. Specimens were deposited in the herbarium of the Kunming Institute of Botany Academia Sinica (HKAS), while living cultures (2 tubes of PDA, 2 tubes of double-distilled water (ddH_2_O) and 2 tubes of 10% of Glycerol) were deposited in the Kunming Institute of Botany Culture Collection (KUMCC) separately at −4 °C and −20 °C. Index Fungorum and Fungal Name numbers were registered for new taxa [15,16].

### 2.2. DNA Extraction, PCR Amplification, and Sequencing

Mycelia (50–100 mg) or fruiting bodies were collected from pure culture and woody host by using sterilized needles and transferred to 1.5 mL centrifugal tubes for DNA extraction. The genomic DNA was extracted by following the protocol in the manufacturer of Biospin Fungus Genomic DNA Extraction Kit-BSC14S1 (BioFlux^®^, P.R. China). The extracted DNA was stored at 4 °C for the polymerase chain reaction (PCR) or maintained at −20 °C for long-term storage.

The PCR mixture contains 8.5 µL of double-distilled water (ddH_2_O), 12.5 µL of 2 × Power Taq PCR MasterMix (mixture of EasyTaqTM DNA Polymerase, dNTPs, and optimized buffer, Beijing Bio Teke Corporation (Bio Teke), PR China), 1 µL of each forward and reverse primers (10 pmol) and 2 µL of DNA [13]. The PCR primers ITS4/ITS5, NS1/NS4, and LR0R/LR5 were used to amplify the internal transcribed spacer (ITS), the partial 18S small subunit (SSU), and the large subunit (LSU) genes, respectively [17,18]. Moreover, the partial RNA polymerase II subunit (*rpb2*) region was amplified by the primers fRPB2-5F/ fRPB2-7cR or LasF/LasR, the partial RNA polymerase II subunit (*rpb1*) region was amplified by the primers RPB2-AF/fRPB2-Cr [19,20], the partial translation elongation factor 1-alpha (*tef1-α*) region was amplified with the primers 983F/2218R, 688F/1251R, 728F/986R or 688F/1251R [21,22,23], the calmodulin (CAL) region was amplified by the primers 228F/737R [22], and the beta-tubulin (*tub2*) gene was amplified by the primers Bt2a/Bt2b or T1/T22 [24,25]. The conditions for PCR of ITS, SSU, LSU genes constituted an initial denaturation step of 2 min at 94 °C, followed by 35 cycles of 30 s at 94 °C, 50 s at 55 °C and 1 min at 72 °C, and a final denaturation step of 10 min at 72 °C. For the *rpb1, rpb2* and *tef1-α* genes, the initial denaturation at 95 °C for 3 min; denaturation at 95 °C for 45 s, annealing at 57 °C for 50 s and extending 90s at 72 °C for 35 cycles; and extending at 72 °C for 10 min. For the CAL gene, the initial denaturation at 95 °C for 3 min; denaturation at 95 °C for 30 s, annealing at 54 °C for 50 s and extending 90s at 72 °C for 35 cycles; and extending at 72 °C for 10 min. For the *tub2* genes, the initial denaturation at 95 °C for 3 min; denaturation at 95 °C for 30 s, annealing at 52 °C for 35 s and extending 90s at 72 °C for 35 cycles; and extending at 72 °C for 10 min. The different primers were used to amplify the different genic loci of special fungal groups. The ITS (ITS4/ITS5) and β-tubulin (Bt2a/Bt2b) gene regions were used for the phylogenetic analyses of Diatrypaceae [26]. The LSU(LR0R/LR5), ITS (ITS4/ITS5), SSU (NS1/NS4), *rpb1* (AF/Cr) gene regions were used for phylogenetic analyses of *Cyphellophora* [27]. The ITS (ITS4/ITS5), *tef1-α* (728F/986R), β-tubulin (Bt2a/Bt2b) and CAL (228F/373R) gene regions were used for phylogenetic analyses of *Diaporthe* [28]. The LSU (LR0R/LR5), β-tubulin (T1/T22), ITS (ITS4/ITS5) and *rpb2* (5F/7cR) gene regions were used for phylogenetic analyses of Hypoxylaceae [29]. The ITS (ITS4/ITS5), *tef1-α* (668F/986R), *rpb2* (LasF/LasR) and β-tubulin (Bt2a/Bt2b) gene regions were used for phylogenetic analyses of *Lasiodiplodia* [30]. The LSU (LR0R/LR5), ITS (ITS4/ITS5) and *tef1-α* (688F/1251R) gene regions were used for phylogenetic analyses of *Aplosporella* [31]. The LSU (LR0R/LR5), SSU (NS1/NS4), ITS (ITS4/ITS5) and β-tubulin (Bt2a/Bt2b) gene regions were used for phylogenetic analyses of *Pleurostoma* [32]. PCR products were sent to Beijing Bio Teke Corporation for purification and sequencing. To ensure the accuracy of the sequencing, the above methods were repeated to get extra sequences for the new taxa.

### 2.3. Phylogenetic Analyses

The generated sequence data were assembled using the Geneious (Restricted) 9.1.2 (https://www.geneious.com, accessed on 2 May 2022). The consensus sequences were subjected to BLASTn searches in the nucleotide database of GenBank (www http://blast.ncbi.nlm.nih.gov/, accessed on 2 May 2022) to screen their most probable closely related taxa, and a spreadsheet was made for accession numbers (Tables S1–S7). Each gene sequence alignment was separately made via MAFFT online version 7, using default settings (http://mafft.cbrc.jp/alignment/server, accessed on 2 May 2022) [33] and manually edited in BioEdit 7.2.3 [34]. The uninformative gaps and ambiguous regions were removed by trimAL v1.2 (http://trimal.cgenomics.org, accessed on 2 May 2022), and multi-genes were manually combined in BioEdit. The fasta files were transferred to PHYLIP (for ML) and NEXUS (for BI) formats in Alignment Transformation Environment (ALTER) online program [35]. The maximum likelihood analysis (ML) was generated via RAxML-HPC BlackBox (8.2.4) [36,37] in the CIPRES Science Gateway v.3.3 (http://www.phylo.org/portal2, accessed on 2 May 2022 [38]) with GTRGAMMA substitution model with 1000 bootstrap iterations. The Bayesian analysis was performed by MrBayes on XSEDE (3.2.7a) via the CIPRES Science Gateway V.3.3 web server [38]. Bayesian posterior probabilities (BYPP) [39] were evaluated by Markov Chain Monte Carlo sampling (MCMC). The best models of evolution were estimated by using MrModeltest v. 2.3 [40] and PAUP v. 4.0b10 [41]. Six simultaneous Markov chains were run for 1,000,000 to 10,000,000 generations, depending on individual settings for the fungal group and the resulting trees were sampled at one tree every 1000th generation. Phylogenetic trees were visualized using FigTree v1.4.0 [42] and the trees were edited by Microsoft PowerPoint and inserted with reliable statistical supports from ML and BI.

## 3. Results

### Taxonomy and Phylogenetic Analyses Results

Sordariomycetes O.E. Erikss. & Winka 1997

Xylariales Nannf., Nova Acta Regiae Societatis Scientiarum Upsaliensis 8 (2): 66 (1932)

Diatrypaceae Nitschke, Verh. Naturhist. Vereines Preuss. Rheinland: 73 (1869)

*Mangifericola* E.F. Yang & Tibpromma, gen. nov.

Fungal Name number: FN 571236

Etymology: The name reflects that the genus is closely associated with the host *Mangifera indica*.

Type species: *Mangifericola hongheensis* E.F. Yang & Tibpromma

*Saprobic* on dead terricolous wood. Sexual morph: *Pseudostroma* poorly developed, delimited with a black surface, irregular, and raised. *Ascomata* immersed, irregular to subglobose, clustered, visible emerging apical parts of extended neck on distinct black region. *Ostioles* cylindrical, sulcate, ostiolar canal periphysate. *Peridium* thick near ostiole, multilayers, dark brown-walled outwardly, comprised by hyaline, compressed *textura angularis* cells to inner layers. *Paraphyses* not seen. *Asci* 8-spored, cylindrical to oblong, unitunicate, pedicellate, straight to fairly curved, hyaline, apically flat. *Ascospores* oblong to allantoid, hyaline, or yellowish, with oil droplets at both ends when mature. Asexual morph: Undetermined.

Notes: *Mangifericola* (*M*.) generally fits with the common concept of Diatrypaceae by having ascomata immersed, subglobose or irregular, with elongated neck, hamathecium absent, asci eight-spored, straight to fairly curved, allantoid ascospores, hyaline or yellowish, with oil droplets [43,44]. However, *Mangifericola* is mainly distinguished from other genera in this family by having a single prolonged neck erect from black pigmented lesions on the wood surface. Additionally, this new genus is distinguished from other closely related genera based on phylogenetic analyses (Figure 1). The results from BLASTn searches of ITS, LSU, and β-tubulin are shown in Table 1. Despite the BLASTn results of ribosomal DNA regions of *Mangifericola* indicating it is closely related to *Diatrype* and *Diatrypella*, the β-tubulin gene indicated it is related to *Melanostictus* with a low similarity. *Diatrypella* is characterized by cushion-like or discoid stromata, umbilicate or sulcate ostiolar necks, cylindrical, polysporous, long-stalked asci, and allantoid, hyaline or yellowish ascospores [45]. Our new genus is similar to *Diatrype* by having allantoid, hyaline or yellowish ascospores but differs in having poorly developed pseudostroma, irregular to subglobose ascomata with a single prolonged neck erect from black pigmented and cylindrical to oblong asci. Therefore, we establish *Mangifericola* (Diatrypaceae) as a distinct new genus.

*Mangifericola hongheensis* E.F. Yang & Tibpromma, sp. nov. (Figure 2).

Fungal Name number: FN 571237

Etymology: The name reflects the location “Honghe” where the holotype was collected.

Holotype: HKAS 122665

*Saprobic* on dead branch of *Mangifera indica*. Sexual morph: *Pseudostroma* poorly developed, delimited with a black surface, irregular, and raised. *Ascomata* (exclude neck) 190–230 μm × 300–342 μm (x = 210 × 322 μm, *n* = 10) daim., semi-immersed to immersed beneath host epidermis or raised to erumpent, perithecial, irregular to subglobose, solitary, visible emerging apical parts of extended neck on distinct black region, scattered, glabrous, individual ostiole with a long neck 150–220 × 80–110 µm (x = 185 × 95 μm, *n* = 15), two of third fully immersed, cylindrical, straight to slightly bent. *Ostioles* cylindrical, sulcate, ostiolar canal periphysate. *Peridium* 17–25 µm wide, composed of two layers: outer layer of dark-brown, thick-walled cells; inner layer: hyaline, thin-walled cells forming *textura angularis*. *Paraphyses* not observed. *Asci* 35–50 × 4–6 µm (x = 45 × 5 μm, *n* = 45), 8-spored, unitunicate, thin-walled, clavate to cylindric-clavate, straight to slightly curved, hyaline, with oil droplets, with a “J-” apical apparatus, pedicellate up to 10–14 μm long. *Ascospores* 5.7–7 × 1.4–3.1 µm (x = 6.4 × 2.3 μm, *n* = 15), 2–3 seriate, overlapping, allantoid, aseptate, 1–2 oil droplets, guttulate, smooth-walled. Asexual morph: Undetermined.

Material examined: China, Yunnan Province, Baoshan City, Longling County, on a dead branch of *Mangifera indica* (99°16′80″ E, 25°12′23″ N, Elevation: 800 m) 27 December 2019, E.F. Yang, MB008 (HKAS 122665, holotype), isotype, HKAS 122666. GenBank numbers: HKAS 122665, ITS: OM030351, LSU: OM030346, β-tubulin: ON468664; HKAS 122666, ITS: OM030348, LSU: OM030350, β-tubulin: ON468665.

Notes: *Mangifericola hongheensis* was collected from a dead wood piece of *Mangifera indica*, and it clearly differs from other taxa in the family Diatrypaceae based on multi-gene phylogenetic analyses and morphological comparisons. The new species formed a well-separated clade distant from other genera within Diatrypaceae. Morphologically, our species is relatively similar to *Diatrype palmicola* (MFLU 15-0040, holotype), and they all cause black pigmented lesions on the wood epidermis, ascomata clustered as small groups, fully immersed, visible extended neck raised above, and the absence of hamathecium (Figure 2). However, the ascomata are different by coloration (dark black vs. brown), and the asci of our species have a shorter pedicel [46]. The comparison of the ITS, and LSU bp regions also showed that the two species have big differences in base pairs (ITS: 8.1%; LSU: 3.1% bp differences) [46], but we were not able to compare the β-tubulin gene of *D*. *palmicola* as they lack of β-tubulin gene. In addition, we compared the *M. hongheensis* with the closely related species in phylogeny and from BLASTn results (*Melanostictus* sp., *Pedumispora* sp., *Halodiatrype* sp., *Diatrypella* sp. and *Diatrype* sp.) but they differ. Therefore, based on both unique morphological characteristics and molecular data we establish the new genus *Mangifericola* with *M. hongheensis* as the type species from China.

Hypoxylaceae DC., Flore française 2: 280 (1805)

*Hypoxylon* Bull., Histoire des champignons de la France. I: 168 (1791)

Index Fungorum number: IF2456

Type Species: *Hypoxylon fragiforme* (Pers.) J. Kickx f., Flore cryptogamique des environs de Louvain, ou déscription des plantes cryptogames et agames qui croissent dans le Brabant et dans une partie de la province d’Anvers: 116 (1835)

Notes: *Hypoxylon* (*H*.) was described by Bulliard [47] with the type species *Hypoxylon fragiforme* (Pers.). The generic concept of *Hypoxylon* traditionally differs from other genera in the family Xylariaceae by four main characteristics viz. *Nodulisporium*-like asexual morph; unipartite stromata; solid and homogeneous stromatal tissue below the perithecial layer; and stromata not upright [48,49,50]. Later, the molecular studies led to the segregation of further genera such as; *Annulohypoxylon* [51], *Hypomontagnella* [52], *Jackrogersella*, and *Pyrenopolyporus* [53] which were previously considered *Hypoxylon* taxa. *Hypoxylon* as the type genus accommodates 829 records in Index Fungorum [16]. The members of *Hypoxylon* frequently grow on dead wood as wood degrading fungi, and they are also often isolated as endophytes of seed plants [52,53]. *Hypoxylon* species are an excellent source of bioactive secondary metabolites, e.g., *H. fuscum* [54]. The phylogenetic relationships of this generic species are shown in Figure 3.

**Figure 4:**
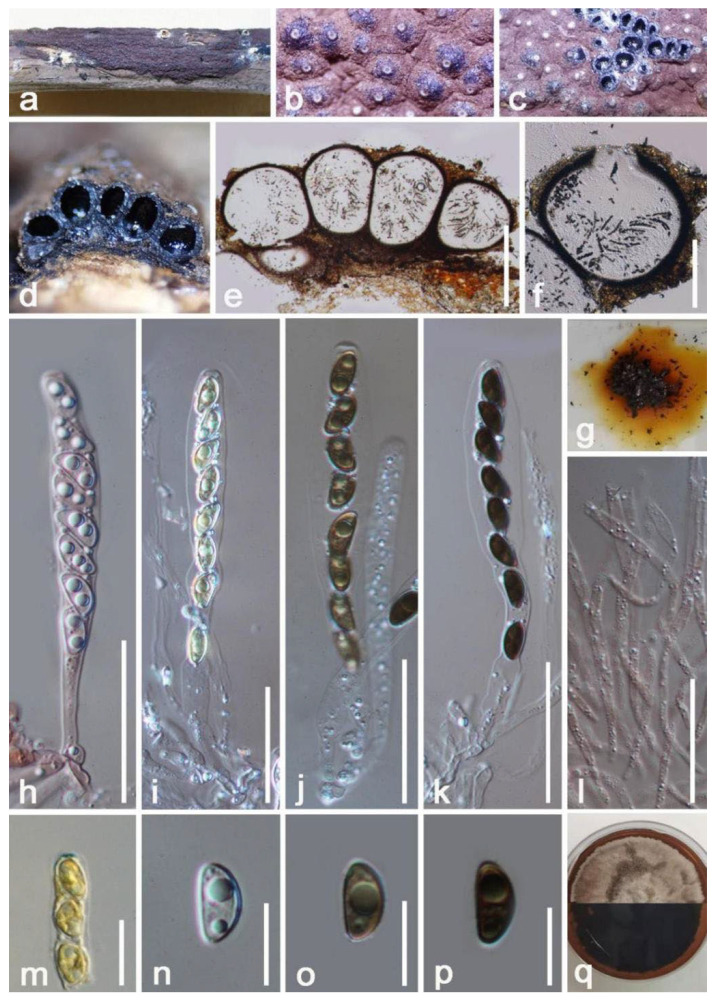
*Hypoxylon hongheensis* (HKAS 122663, holotype). (**a**) Ascomata on the host surface; (**b**) Close-up of ascomata; (**c**) Horizontal section of ascomata; (**d**–**f**) Vertical section through ascomata; (**g**) Pigments of ascomata in 10% KOH solution; (**h**–**k**) Asci (**h** asci stained by Congo red reagent); (**l**) Paraphyses stained by Congo red reagent; (**m**) “J+” apical apparatus. (**n**–**p**) Ascospores; (**q**) Colony on PDA. Scale bars: (**e**,**f**) = 300 μm; (**h**–**l**) = 15 μm; (**i**–**j**) = 10 μm.

*Hypoxylon hongheensis* E.F. Yang & Tibpromma, sp. nov. (Figure 4).

Index Fungorum number: IF 559413

Etymology: The name reflects the location “Honghe” where the holotype was collected.

Holotype: HKAS 122663

*Saprobic* on dead branch of *Mangifera indica*. Sexual morph: *Stromata* 300–500 μm high, effused-pulvinate, superficial, surface reddish-brown to brown, tough, raised, multiloculate, composed of carbonaceous tissue-like and conspicuous brownish black, with 10% KOH extractable pigments dark orange. *Perithecia* 300–500 μm (x = 400 μm, *n* = 10) high, 280–450 μm (x = 360 μm, *n* = 10) wide, spherical to obovoid, immersed in carbonaceous tissue, arranged as multi-layers. *Ostioles* central, white, sunken, whitish-granulate surrounded margin, opened when stromata well-developed, short cylindrical, around at the same level of the astromatal surface, ostiolar canal 47–59 μm high, 80–91 μm wide. *Peridium* 21–30 µm wide, multi-layers, composed of brown thick-walled cells of *textura angularis*, inner layers with hyaline, equal thickness, dark-brown to black. *Hamathecium* 1.8–3 μm wide, filamentous, anastomosis, cylindrical, aseptate, unbranched, with granules, paraphyses. *Asci* 67–102 × 7–11 μm (x = 85 × 9 μm, *n* = 20), 8-spored, unitunicate, cylindrical, hyaline, oblong, apically rounded, medium pedicellate, with pedicel 16–22 µm wide, with a “J+” apical apparatus. *Ascospores* 9–11 × 4.5–5.5 μm (x = 11 × 5 μm, *n* = 20), uniseriate, crescent to somewhat hemispherical, at first hyaline to yellow becoming brown or black when mature, usually 1–2 guttules, aseptate, rounded at the ends, without germ slit or gelatinous sheath or appendages. Asexual morph: Undetermined.

Culture characteristics: Colonies on PDA 50 mm in diameter after two weeks at 27 °C, gray, after around two months, colonies on PDA becoming reddish-brown, circular, regular margin, flat; dark brown at the reverse, with reddish pigments produced in PDA. No sporulation on PDA and oatmeal agar (OMA) media within three months, Vegetative hyphae 2-4 μm wide, hyaline, smooth-walled.

Material examined: China, Yunnan Province, Honghe Menglong Village, on a dead branch of *Mangifera indica*, (102°50′11″ E, 23°41′01″ N, 500 m), 24 July 2019, E.F. Yang, EFH003 (HKAS 122663, holotype), ex-type living culture KUMCC 21-0452. GenBank numbers: HKAS 122663, ITS: OM001336, LSU: OM001339, β-tubulin: ON468656, *rpb2*: ON392009; KUMCC 21-0452, ITS: OM001333, LSU: OM001334, β-tubulin: ON468655, *rpb2*: ON39008.

Notes: Based on morphology, our isolates fit with the concept of *Hypoxylon* by having effused-pulvinate, unipartite ascomata, with solid and homogeneous, stromatal tissue. The BLASTn results for ITS, *rpb2*, β-tubulin, and LSU region are shown in Table 1. In addition, the phylogenetic analysis of combined LSU, ITS, *rpb2* and β-tubulin sequence showed our strains (HKAS 122663, KUMCC 21-0452) separate well from *H. perforatum* (CBS 115281) (Figure 3). Following the description of *H. perforatum* by Khodaparast [55] and Kout & Zíbarová [56] we compared the asci and ascomatal pigments in 10% KOH solution and indicated the differences with *H*. *perforatum*, viz. (asci: 80–125 × 5–8.7 µm vs. 67–102 × 7–11 μm; ascomatal pigments: yellowish vs. dark orange to reddish). Therefore, we introduce our strain as a new species (*H. hongheensis*) based on morphology and phylogenetic evidence.

Diaporthales Nannf., Nova Acta R. Soc. Scient. upsal., Ser. 4 8(no. 2): 53 (1932)

Diaporthaceae Höhn. ex Wehm., American Journal of Botany 13: 638 (1926)

*Diaporthe* Nitschke, Pyrenomycetes Germanici 2: 240 (1870)

Index Fungorum number: IF 172054

Type species: *Diaporthe eres* Nitschke, Pyrenomycetes Germanici 2: 245 (1870)

Notes: *Diaporthe* (*D*.) was established by Nitschke [57] with the type species *D*. *eres*, and it was placed in the family Diaporthaceae [58]. Previously, taxa in this genus were known as the asexual morph and named as *Phomopsis*, but it was replaced by the sexual morph typified name *Diaporthe* [59]. The species identification of *Diaporthe* spp. is traditionally based on host association and phenotypic features, and the sexual morph is characterized by having immersed ascomata, with erumpent pseudostroma, fusoid, ellipsoid to cylindrical, hyaline ascospores, with or without septate, and sometimes having appendages [57,60], while the asexual morph produces three kinds of conidia viz. α-conidia (straight, guttulate or eguttulate, smooth-walled), β-conidia (straight or hamate, smooth-walled, guttulate), and γ-conidia (seldom produced, multiguttulate, fusiform to subcylindrical, apically acute or rounded while the base is sometimes truncate) [61]. *Diaporthe* species have been reported occurring on various plants as pathogens, saprobes, or endophytes worldwide, and in addition, *Diaporthe* is responsible for many diseases such as root and fruit rot, dieback, cankers, leaf spot, leaf and pod blights, wilt, and seed decay of economically important agricultural crops or woody hosts [62]. Additionally, *Diaporthe* species have been reported as pathogens associated with humans and other mammals [63]. *Diaporthe* also has the potential to stop herbivory by lignocellulolytic activities [64], and Ash et al. [65] reported its use as a bioherbicide. The phylogeny of this genus is shown in Figure 5.

**Figure 6:**
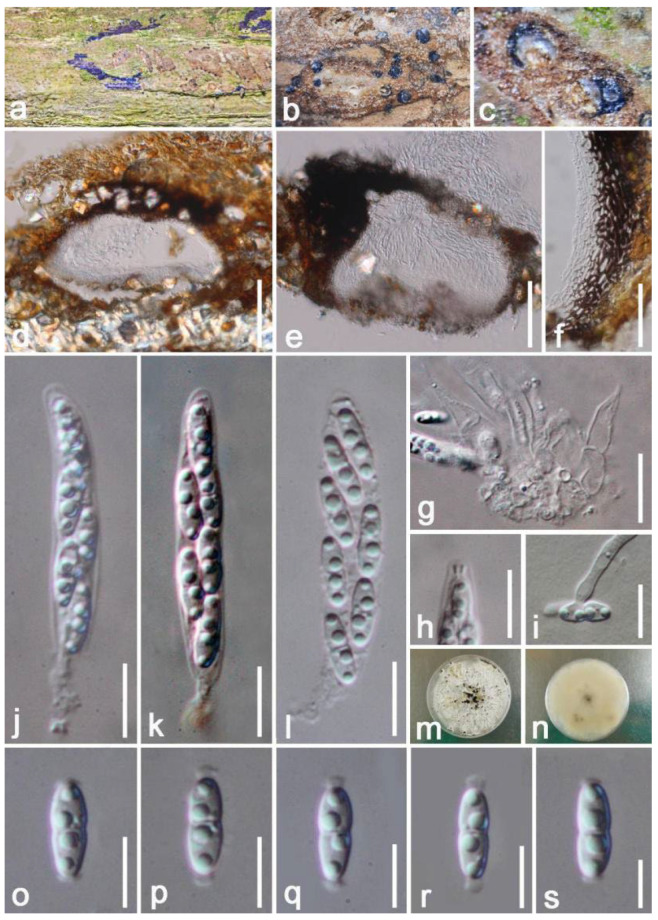
*Diaporthe hongheensis* (HKAS 122657, holotype). (**a**) Appearance of ascomata on the host; (**b**,**c**) Close-up of ascomata with horizontal section; (**d**,**e**) Sections through of ascomata; (**f**) Peridium; (**j**–**l**) Asci ((**k**), stained by Congo red reagent); (**g**) Paraphyses; (**h**) apical apparatus; (**i**) Ascospore with germinated tube; (**m**,**n**) Colony on PDA; (**o**–**s**) Ascospore. Scale bars: (**d**,**e**) = 100 μm; (**f**) = 35 μm; (**g**) = 15 μm; (**h**–**l**) = 10 μm; (**o**–**s**) = 5 μm.

*Diaporthe hongheensis* E.F. Yang & Tibpromma, sp. nov. (Figure 6)

Index Fungorum number: IF 559411

Etymology: The name reflects the location “Honghe” where the holotype was collected.

Holotype: HKAS 122657

*Saprobic* on dead branch of *Mangifera indica*. Sexual morph: *Ascomata* perithecial 120–190 × 250–340 μm (x = 155 × 295 μm, *n* = 20), completely immersed under clypeus, subglobose to oval, brown to dark brown, solitary to gregarious, smooth-walled, non-papillate, ostiole. *Peridium* 19–33 μm wide, multilayer, dark brown to black thick-walled cells, and the inner layers comprised of flattened, hyaline cells of *textura prismatica*. *Hamathecium* 4–7 μm wide, broadly cylindrical, thick-walled, septate, unbranched, attached with a gelatinous matrix, psudoparaphyses. *Asci* 43–51 μm × 5–7 μm (x = 47 × 6 μm, *n* = 20), 6(–8)-spored, cylindrical, hyaline, unitunicate, with an amyloid ring, short pedicellate, apex rounded. *Ascospores* 9–10 × 2–4 μm (x = 9.5 × 3 μm *n* = 20), overlapping uniseriate, fusiform, 1-septate, constricted at septa, hyaline, round ends with two polar appendages, smooth-walled, normally appear 4 droplets. Asexual morph: Undetermined.

Culture characteristics: Colonies on PDA 50–70 mm in diameter after two weeks at 27 °C, white to gray, circular, flat to effuse, medium dense, fimbriate margin; white at the reverse, without pigments produced in PDA, but produced black dots and released fluid secretions after one month, but without any spores were observed. Vegetative hyphae 1–3 μm wide, hyaline, smooth-walled.

Material examined: China, Yunnan Province, Honghe, on a dead branch of *Mangifera indica* (102°50′11″ E, 23°41′01″ N, 800 m) 22 December 2020, E.F. Yang, HHE011 (HKAS 122657, holotype), ex-type living culture KUMCC 21-0457 = KUMCC 21-0458. GenBank numbers: KUMCC 21-0457, ITS: OM001331, β-tubulin: ON468658, CAL: ON715010, *tef1-α*: ON468649; KUMCC 21-0458, ITS: OM001330, β-tubulin: ON468659, CAL: ON715009, *tef1-α*: ON468650.

Notes: *Diaporthe pseudomangiferae* and *D*. *pseudophoenicicola* were isolated from *Mangifera indica* with or without pathogenetic symptoms [63]. Our new isolate *D*. *hongheensis* fits well with the concept of *Diaporthe* by having fully immersed, mostly subglobose, ellipsoid to cylindrical ascomata, hyaline, septate ascospores with appendages [58]. *Diaporthe hongheensis* is different by having 6(–8)-spores asci, while most *Diaporthe* species which available sexual morph have 8-spored asci, like *D*. *eucalyptorum*, *D*. *alnea,* and *D. neilliae* [67]. The BLASTn results of ITS, β-tubulin, CAL, and *tef1-α* gene region were shown in Table 1. In addition, the multi-locus phylogenetic analysis separated *D*. *hongheensis* (KUMCC 21-0457) well although closely related to *D*. *viniferae, D*. *pandanicola* and *D*. *fraxini-angustifoliae* clades. In addition, for those phylogenetically closely related species, only asexual morphs are available. Therefore, we introduce *D*. *hongheensis* as novel species based on morpho-molecular analyses.

Eurotiomycetes O.E. Erikss. & Winka 1997

Chaetothyriales M.E. Barr, Mycotaxon 29: 502 (1987)

Cyphellophoraceae Réblová & Unter., PLoS One 8 (5): 10 (2013)

*Cyphellophora* G.A. de Vries, Mycopathologia et Mycologia Applicata 16: 47 (1962)

Index Fungorum number: 7885

Type species: *Cyphellophora laciniata* G.A. de Vries, Mycopathologia et Mycologia Applicata 16: 47 (1962)

**Figure 7:**
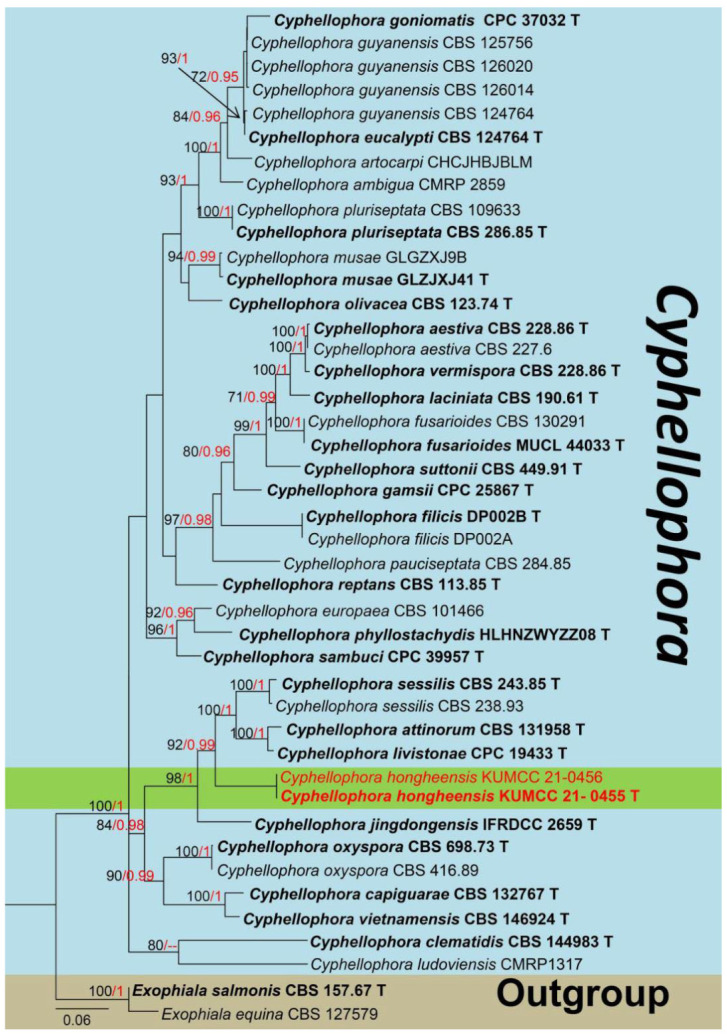
Phylogram of a new species *Cyphellophora hongheensis* and other species in genus *Cyphellophora* generated from maximum likelihood analysis based on a combined LSU, ITS, SSU, *rpb1* sequence datasets, with *Exophiala equina* (CBS 127579) and *Exophiala salmonis* (CBS 157.67) as the outgroups. Related sequences used in the phylogeny were taken from Crous et al. [27]. The species introduced in this study are indicated in red, and the type strains are indicated in bold with “T”. Bootstrap values equal to or greater than 70% (ML, Left) and Bayesian posterior probabilities (BI, right) equal to or greater than 0.95 are given at the nodes. Hyphens (-) represent values less than 70% in ML/0.95 in BI. For more information, please see the Supplementary Materials (Table S4, Supplementary information S4).

Notes: The genus *Cyphellophora* (*C*.) with *C. laciniata* as the type species was established in 1962 [68]. To date, *Cyphellophora* contains a total of 31 species excluding three species that have been transferred to *Aphanophora, Camptophora,* and *Pseudomicrodochium* [46]. The members of *Cyphellophora* species are usually reported as saprobic on excretions of insects or foliar epiphytes on living leaves worldwide, and also as endophytic fungi on fresh leaves, while Yang et al. [69] introduced the first sexual morph record with the new species *C. jingdongensi* (IFRD 9049) from living leaves of *Alnus nepalensis* in China. In addition, *C*. *laciniata*, *C. europaea* and *C*. *pluriseptata* were found associated with human and animal skin and nails [70]. *Cyphellophora sessilis* was reported as a pathogenic fungus that causes sooty blotch, flyspeck, and diseases of certain fruit corps [71]. The sexual morph of *Cyphellophora* is characterized by ascomata fusing with host tissue at the base, scattered, subglobose to globose, dark brown, and ostiole inconspicuous; asci each ellipsoidal to cylindrical, short pedicel, bitunicate, hyaline, and septate ascospores. Based on a previous study, the asexual morph of this genus was described by producing branched hyphae, intercalary, terminal, or lateral, sparse, or integrated; conidiogenous cells phialidic, hyaline or pale brown, conidia ranging in shape from oblong to fusiform or vermiform [70]. The phylogeny of this genus is shown in Figure 7.

**Figure 8:**
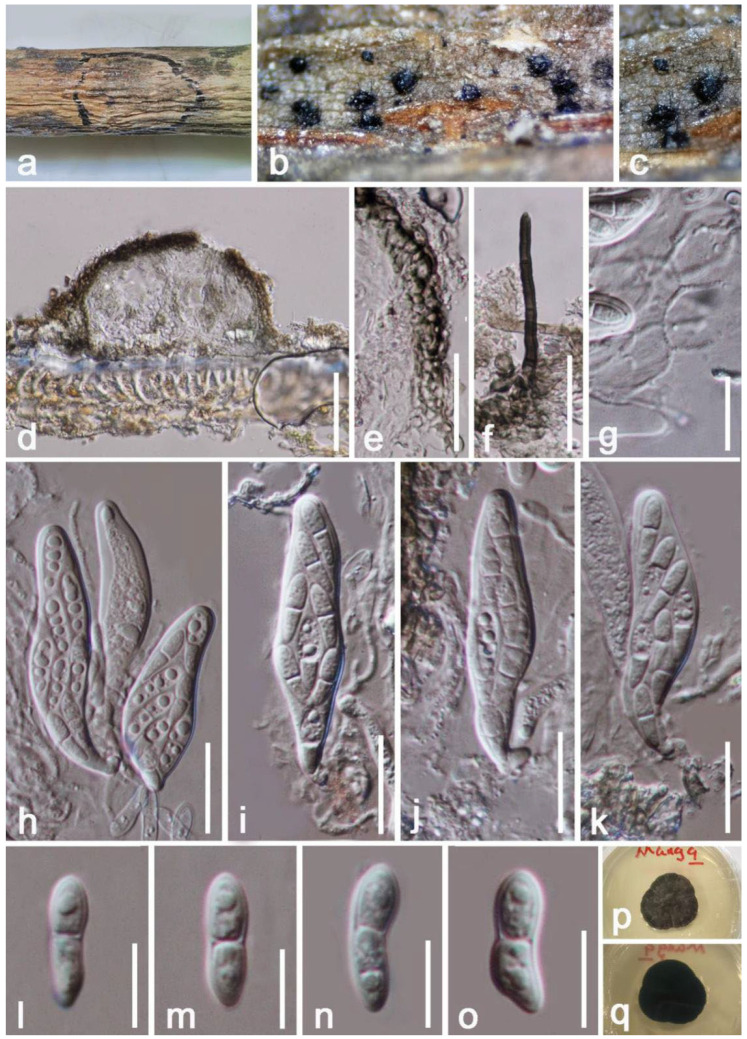
*Cyphellophora hongheensis* (HKAS 122661, holotype). (**a**) Ascomata on decaying wood of mango; (**b**–**c**) Close-up of ascomata on substrate; (**d**) Horizontal section of ascomata; (**e**) Peridium; (**f**) Seta; (**g**) Pseudoparaphyses; (**h**–**k**) Asci; (**l**–**o**) Ascospores; (**p**) Obverse view of colony on PDA; (**q**) Reverse view of colony on PDA. Scale bars: (**d**) = 50 μm; (**f**) = 30 μm; (**e**) = 25 μm; (**h**–**k**) = 20 μm; (**g**) =15 μm; (**l**–**o**) = 10 μm.

*Cyphellophora hongheensis* E.F. Yang & Tibpromma, sp. nov. (Figure 8)

Index Fungorum number: IF 559412

Etymology: The name reflects the location “Honghe” from where the holotype was collected.

Holotype: HKAS 122661

*Epiphytic* on a living branch of *Mangifera indica*. Sexual morph: *Ascomata* perithecial 74–102 × 112–147 μm (x = 88 × 130 μm, *n* = 20), scattered to gregarious, superficial, fuse with host tissue at the base, visible as black spots on host, uni-loculate, globose to subglobose, setose, indistinct ostiole. *Setae* 2.5–4 µm wide, 40–70 µm long, black, septate, cylindrical with obtuse apex. *Peridium* 13–19 µm wide, thin-walled cells arranged in *textura globulosa* to *textura angularis*, brown to black. *Hamathecium* 1–1.5 wide, filiform, septate, hyaline, moderately dense, trabeculate, anastomosing, branched, pseudoparaphyses. *Asci* 56–73 × 8–12 μm (x = 64 × 10 μm, *n* = 20), 8-spored, bitunicate, oblong, broadly subcylindrical, very short pedicellate or sometimes disappeared, apically rounded, poorly developed ocular chamber, straight to mildly bent. *Ascospores* 12–16 × 3–5 µm (x =14 × 4 µm, *n* = 20), ellipsoid, overlapping 2–3-seriate, smooth-walled, thick-walled, 1-septate, obtuse ends, slightly constricted at the septum, slightly curved, relatively wider at upper than lower, guttulate, without a mucilaginous sheath. Asexual morph: Undetermined.

Culture characteristics: Colonies on PDA 15–20 mm in diameter after two weeks at 27 °C, brown to black, effuse, circular, dense and rough at the surface, well-defined, undulate edge with sinking, slightly striated; dark brown at the reverse, without pigments produced in PDA. Vegetative hyphae 2–4 μm wide, hyaline, branched, septate.

Material examined: China, Yunnan Province, Honghe Menglong Village, on a living branch of *Mangifera indica* without showing any pathogenic symptoms, (102°50′11″ E, 23°41′01″ N, 500 m), 22 December 2020, E.F. Yang, mang9 (HKAS 122661, holotype), ex-type living culture KUMCC 21-0455 = KUMCC 21-0456. GenBank numbers: KUMCC 21-0455, ITS: OM001338, LSU: OM001335, SSU: OM001340, *rpb1*: ON468646; KUMCC 21-0456, ITS: OM001332, LSU: OM001329, SSU: OM001337, *rpb1*: ON468647.

Notes: Our isolates clustered within *Cyphellophora*, while they share similar characteristics with *C*. *jingdongensi* (IFRD 9049) which was reported by Yang et al. [69] from China, but our strains differ by having distinct setae at the outermost of peridium, and ellipsoid to irregular, 1-septate, thick-walled, rough, and smaller (12–16 × 3–5 µm vs. 16–24 × 5–7 μm) ascospores. The BLASTn values and percent-sequence of ITS, SSU, LSU and *rpb1* showed that our strain is closely related to the taxa in Table 1, and phylogenetic results indicated that they are separated. Phylogenetic results indicated that our isolates are well separated from *C. attinorum* (CBS 131958), *C. sessilis* (CBS 238.93, CBS 243.85) and *C. jingdongensi* (IFRD 9049) clades with high statistical supports (92% in ML, 0.99 in BI) (Figure 7). Therefore, based on the evidence of morphology and phylogeny, we introduce our strains as a new species, *C. hongheensis* on *Mangifera indica* from China.

Known species

Dothideomycetes O.E. Erikss. & Winka 1997

Botryosphaeriales C.L. Schoch, Crous & Shoemaker, Mycologia 98 (6): 1050 (2007)

Botryosphaeriaceae Theiss. & P. Syd., Annales Mycologici 16 (1-2): 16 (1918)

*Lasiodiplodia* Ellis & Everh., Botanical Gazette Crawfordsville 21: 92 (1896)

Index Fungorum number: IF 8708

Type species: *Lasiodiplodia tubericola* Ellis & Everh., Botanical Gazette Crawfordsville 21: 92 (1896)

**Figure 9:**
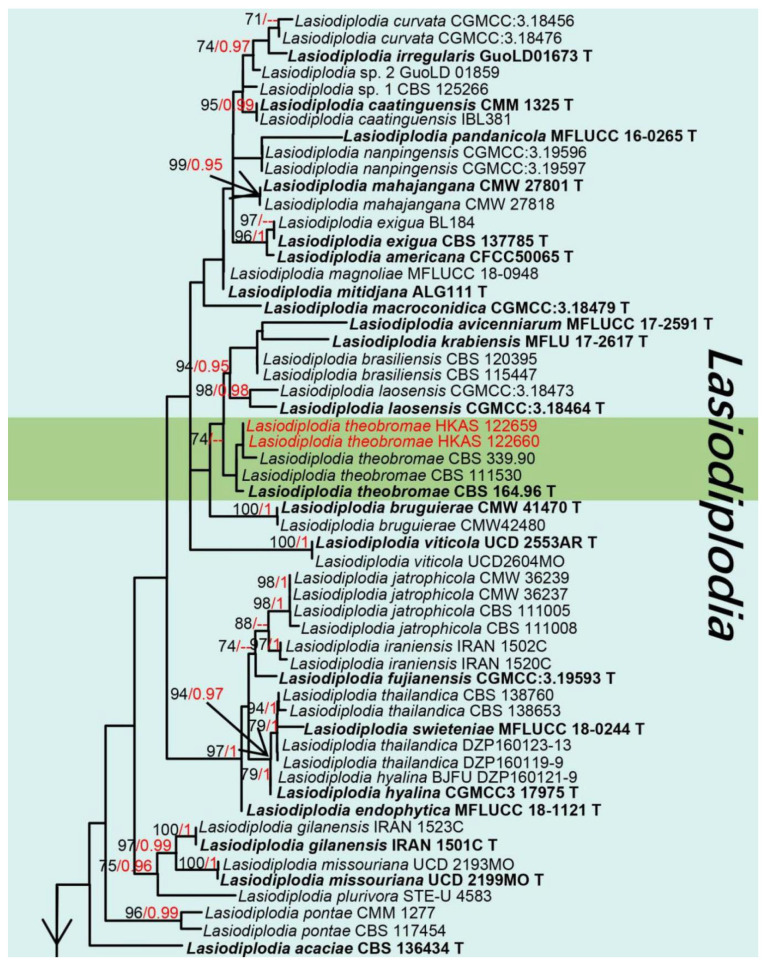

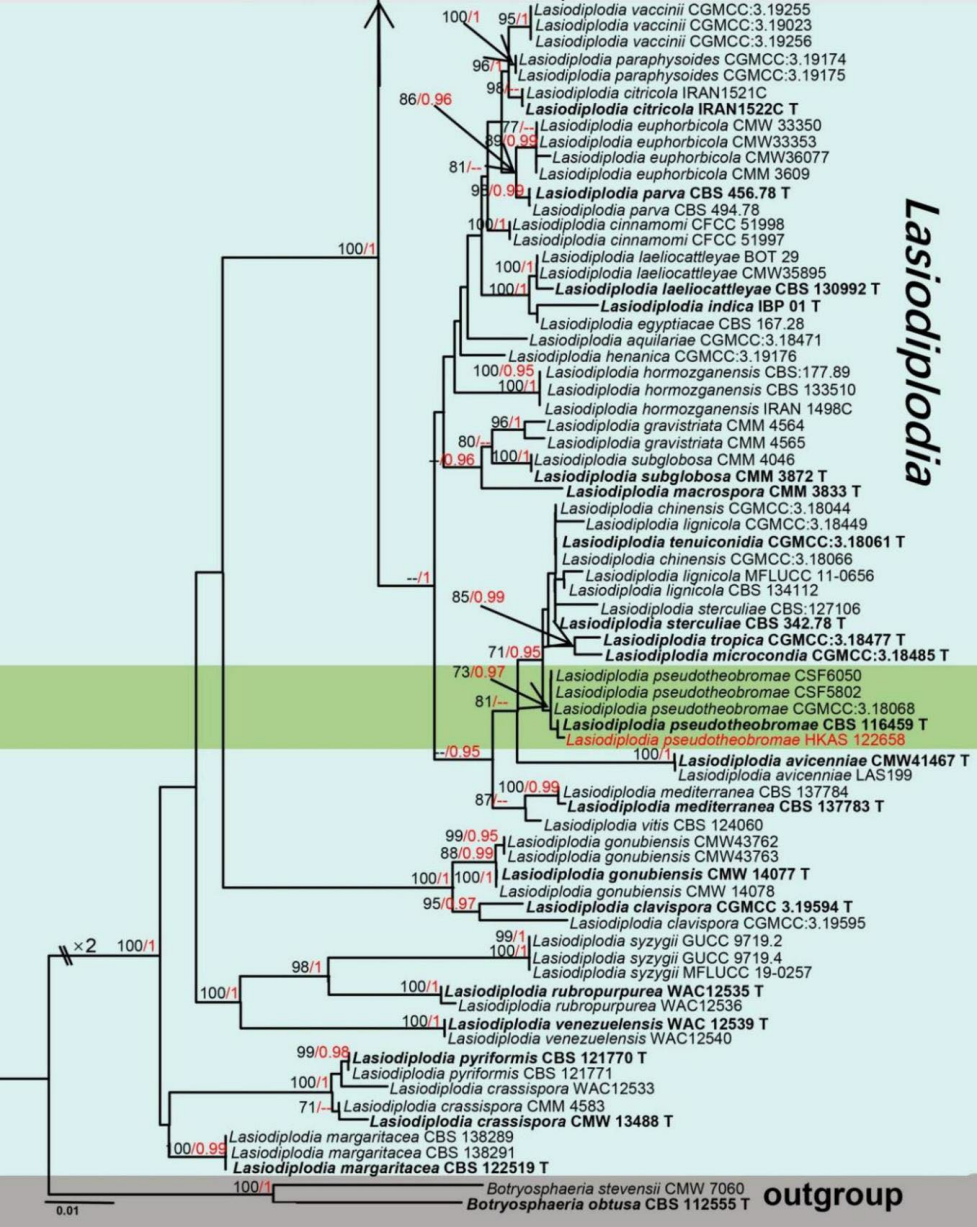
Phylogram of new collections of *Lasiodiplodia theobromae* and *L*. *pseudotheobromae* and related species within the genus *Lasiodiplodia* generated from maximum likelihood analysis based on a combined ITS, β-tubulin and *tef1-α* sequence datasets, with *Botryosphaeria stevensii* (CMW 7060) and *Botryosphaeria obtusa* (CBS 112555) as the outgroups. Related sequences used in the phylogeny were taken from Zhang et al. [30]. The species introduced in this study are indicated in red, and the type strains are indicated in bold with “T”. Bootstrap values equal to or greater than 70% (ML, Left) and Bayesian posterior probabilities (BI, right) equal to or greater than 0.95 are given at the nodes. Hyphens (-) represent values less than 70% in ML/0.95 in BI. For more information, please see the Supplementary Materials (Table S5, Supplementary information S5).

Notes: *Lasiodiplodia* (*L*.) was established by Ellis & Everh [72] with *L. tubericola* as the type species, and to date, this genus contains 69 records in Index Fungorum [16]. Sexual morph of *Lasiodiplodia* species have been poorly documented, but the known sexual morph of *L. gonubiensis, L. lignicola, L. theobromae,* and *L. pseudotheobromae* are characterized by producing ellipsoidal to fusiform, aseptate, straight to curved ascospores, hyaline to brown when mature [73]. The asexual morph of *Lasiodiplodia* was characterized by having pycnidial paraphyses and longitudinal striations on the mature conidia, but identification of *Lasiodiplodia* spp. only relying on main morphological features (conidia and paraphyses) is impossible. Thus, ITS and *tef1-α* regions have been widely used to distinguish different species in this genus [74]. This genus is commonly distributed in subtropical to tropical regions where the temperature is high and is associated with various diseases of woody hosts such as stem blight and/or canker, and dieback [74]. Especially, some *Lasiodiplodia* spp. (*L*. *iraniensis, L*. *theobromae,* and *L*. *laeliocattleyae*) and other species in Botryosphaeriaceae are well-known pathogens associated with mango [75,76]. The phylogeny of *Lasiodiplodia* and closely related genera are shown in Figure 9.

**Figure 10:**
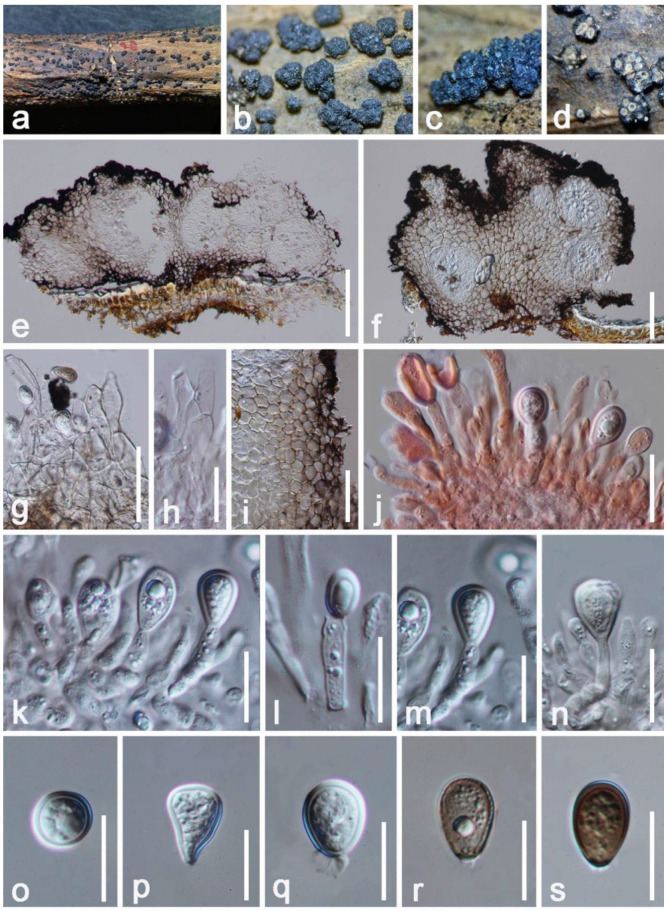
*Lasiodiplodia theobromae* (HKAS 122660). (**a**–**c**) Conidiomata on decaying brunch of *Mangifera indica*; (**d**) Horizontal section of conidiomata; (**e**,**f**) Vertical section of conidiomata; (**g**,**h**) Hamathecium; (**i**) Pycnidial wall; (**j**) Conidiogenous cells with developing conidia were strained by Congo red reagent; (**k**–**n**) Conidiogenous cells with developing conidia; (**o**–**s**) Conidia. Scale bars: (**e**) = 150 μm; (**f**)= 100 μm; (**g**,**i**) = 50 μm; (**n**,**j**) =20; (**h**,**k**–**m**,**o**–**s**) = 15 μm.

*Lasiodiplodia theobromae* (Pat.) Griffon & Maubl., Bull. Soc. mycol. Fr. 25: 57 (1909) (Figure 10)

Index Fungorum number: IF 188476

*Saprobic* on decaying branch of *Mangifera indica*. Sexual morph: Undetermined. Asexual morph: *Stromata* 230–340 × 480–760 μm (x = 285 × 680 μm, *n* = 10), solitary, irregular, rough-walled, superficial, dark brown, scattered, conspicuous on host surface, dull. *Conidiomata pycnidial* 245–350 × 200–280 μm (x = 290 × 220 μm, *n* = 10), globose to subglobose, multi-loculate, brown, without ostiole and papilla. *Pycnidial walls* 50–90 μm (x = 72 μm, *n* = 20) wide, composed of several layers of *textura angularis*, thick-walled, pale brown to dark brown from outside to inward. *Paraphyses* 8–15 μm (x = 12 μm, *n* = 20) wide, cylindrical, smooth and thick-walled, unbranched, hyaline, septate. *Conidiophore* absent. *Conidiogenous cell* 13–18 × 3–5 μm (x = 16 × 4.5 μm, *n* = 20), holoblastic, hyaline, cylindrical to subcylindrical, longed, erect, granulate, straight to slight flexuous. *Conidia* 13–18 × 8–12 μm (x = 16 × 10 μm, *n* = 20) subglobose to obovoid, initially hyaline and later become brown when at mature, aseptate, thick-walled, wall < 3 μm, without longitudinal striations, with abundant oil droplets, round at the apex.

Substrata: *Koelreuteria bipinnata* var. *integrifoliola* [77]; *Mangifera indica* [78,79], (this study).

Distribution: China [77,79], (this study); Africa [78].

Material examined: China, Yunnan Province, Honghe Menglong Village, on a decaying branch of *Mangifera indica*, (102°50′11″ E, 23°41′01″ N, 500 m), 22 December 2020, E.F. Yang, HHE026 (HKAS 122659, HKAS 122660). GenBank numbers: HKAS 122659, ITS: OM030345, LSU: OM030344, *tef1-α*: ON468653, *rpb2*: ON392013; β-tubulin: ON468662. HKAS 122660, ITS: OM030349, LSU: OM030347, *tef1-α*: ON468652, *rpb2*: ON392012; β-tubulin: ON468661.

Notes: Species of *Lasiodiplodia* are mostly distinguished by the morphology of the conidia and paraphyses [74], and our isolate *L. theobromae* fits with the genetic concept of *Lasiodiplodia* by having hyaline to brown, thick-walled conidia, and pycnidial paraphyses. The BLASTn results of *tef1-α*, *rpb2*, ITS, β-tubulin and LSU showed that our isolate is closely related to *L. theobromae* strains with relatively high similarity (100%) (Table 1). In addition, the multi-gene phylogenetic analysis also showed our strains (HKAS 122660 and HKAS 122659) were relatively closely related to *L. theobromae* (CBS 111530, CBS 339.90, and CBS 146.96) (Figure 9). Therefore, *L*. *theobromae* is reported as an extra collection from China and associated with mango.

**Figure 11:**
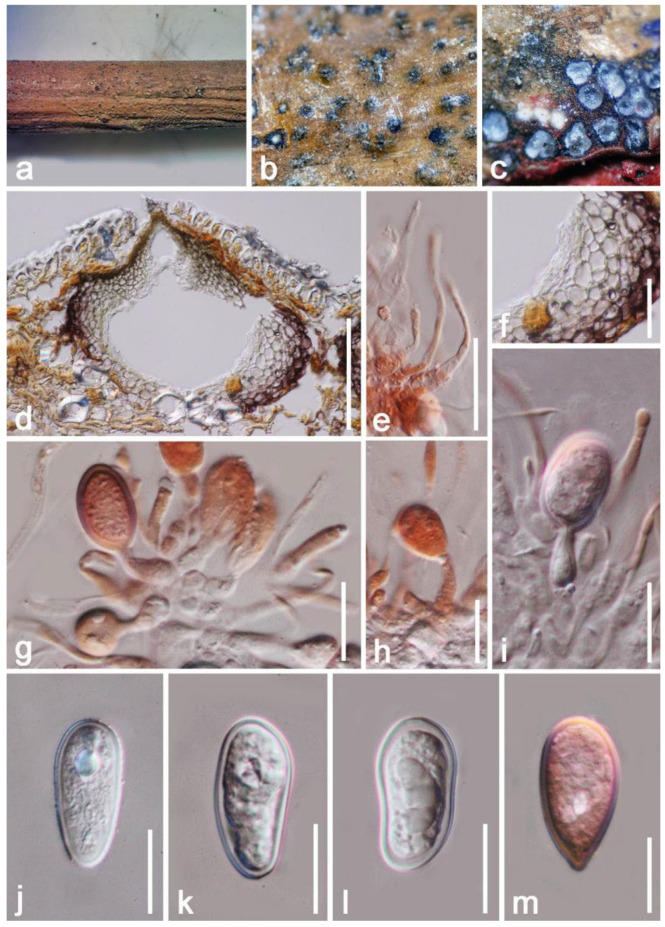
*Lasiodiplodia pseudotheobromae* (HKAS 122658) (**a**,**b**) Conidiomata formed on dead branch of *Mangifera indica*; (**c**) Horizontal section of conidiomata; (**d**) Vertical section of conidiomata; (**e**) Hamathecium stained by Congo red reagent; (**f**) Peridium; (**g**–**i**) Conidiogenous cells with developing conidia stained by Congo red reagent; (**j**–**l**) Conidia; (**m**) Conidia stained by Congo red reagent. Scale bars: (**d**) = 100 μm; (**e**,**f**) = 30 μm; (**g**–**m**) = 15 μm.

*Lasiodiplodia pseudotheobromae* A.J.L. Phillips, A. Alves & Crous, Fungal Diversity 28: 8 (2008) (Figure 11)

Index Fungorum number: IF 510941

*Saprobic* on dead branch of *Mangifera indica*. Sexual morph: Undetermined. Asexual morph: *Conidiomata pycnidial* 130–180 × 170–240 μm (x = 155 × 205 μm, *n* = 20), semi-immersed to totally immersed, ampulliform, solitary to gregarious, dark brown, short ostiole, visible apical black region raised on the top surface. *Ostioles* 43–54 × 57–85 μm (x = 48 × 71 μm, *n* = 10), single, cylindrical with conical at the apical, brown. *Pycnidial walls* 23–55 μm (x = 39 μm, *n* = 20) wide, thick-walled, unequal thickness, the outer layers comprised by brown to dark brown cells of *textura globulosa,* the inner layers comprised of hyaline to brownish cells of *textura angularis*. *Hamathecium* 3–4 μm (x = 3.5 μm, *n* = 20) wide, cylindrical, hyaline, aseptate, wide at the base, unbranched, round at tip, raised among conidia, paraphyses. *Conidiophores* reduced to conidiogenous cells. *Conidiogenous cells* 14–21 × 3–5 μm (x = 17 × 4 µm, *n* = 20), holoblastic, cylindrical to subcylindrical, hyaline, granules, thick-walled, some slightly bent, producing a single conidium at the top. *Conidia* 22–28 × 9–14 μm (x = 25 × 11 μm *n* = 20), obovoid to ellipsoid, hyaline, rounded at the apex, some constricted in the middle, verruculose, granules, thick-walled, wall < 2 μm, hyaline, aseptate, without longitudinal striations or mucilaginous sheath.

Substrata: *Mangifera sylvatica* [80]; *Syzygium* sp. [81]; *Acacia mangium* [82]; *Camellia sinensis* [83]; *Vaccinium* sp. [84]; *Eucalyptus* sp. [85]; *Mangifera indica* ([78], this study).

Distribution: Australia [84]; South Africa [81]; Costa Rica [82]; Thailand [78]; China ([80,83,85], this study).

Material examined: China, Yunnan Province, Honghe Menglong Village, on a dead branch of *Mangifera indica*, (102°50′11″ E, 23°41′01″ N, 500 m), 24 July 2019, E.F. Yang, EFH002-1, (HKAS 122658). GenBank numbers: ITS: OL989258, LSU: OL989296, *tef1-α*: ON468648, *rpb2*: ON392007, β-tubulin: ON468654.

Notes: *Lasiodiplodia pseudotheobromae* was first introduced by Alves et al. [23], and it was established based on immersed, uniloculate, black conidiomata formed on poplar twigs in culture pycnidial, paraphyses hyaline, cylindrical to oblong, sometimes aseptate and branched, blunt ends, conidia ellipsoidal, thick-walled, initially hyaline and aseptate, becoming dark brown with single septum after released. Our collection of *L*. *pseudotheobromae* (HKAS 122658) fully matches the above concept of *L*. *pseudotheobromae*, especially the dimensions of conidia (22–28 × 9–14 μm vs. 27–29 × 15–17 µm). Moreover, the BLASTn and multi-gene analyses (ITS, LSU, *tef1-α*, *rpb2*, β-tubulin) showed that our collection highly overlaps (100%) with *L*. *pseudotheobromae* (Table 1). The phylogenetic analyses also confirmed our strain (HKAS 122658) clusters within *L*. *pseudotheobromae* strains with high statistical support (Figure 9, 85% in ML, 1 in BI). Therefore, our collection is reported as *L*. *pseudotheobromae* which was associated with mango.

Aplosporellaceae Slippers, Boissin & Crous, Stud. Mycol. 76(1): 41 (2013)

*Aplosporella* Speg., Anales de la Sociedad Científica Argentina 10: 157 (1880)

Index Fungorum number: IF 7191

Type species: *Aplosporella chlorostroma* Speg., Anales de la Sociedad Científica Argentina 10: 158 (1880)

Notes: *Aplosporella* (*A*.) was first introduced by Spegazzini [86], with *A*. *chlorostroma* as the type species. The members of this genus are frequently found as saprophytic or parasitic on various plants worldwide, and most country records were concentrated in India (41.23% of 456 total records) based on the U.S. National Fungus Collections Fungal Database [8]. The sexual *Aplosporella* was identified by having solitary to small group ascomata, erumpent from host epidermis, pulvinate, multi-loculate, locules rectangular, numerous asci in each locule, peridium comprised by thin-walled, black cells of *textura angularis*, 8-spored asci, cylindric-clavate, short pedicellate, round apex, with a distinct apical chamber. The ascospores were hyaline, ellipsoid to ovate, smooth, thick-walled, and arranged in 1–2 series in each ascus [87]. Asexual morph of *Aplosporella* is characterized by immersed, or bursts out of the bark, subglobose, multiloculate conidiomata, with a single ostiolar canal, cylindrical, hyaline conidiophores producing brown, aseptate, slightly verruculose conidia [88]. Most species of *Aplosporella* are presently known to have been associated with small, dead twigs, and are already reported as pathogens, endophytes, and saprobes on various plants (including mango) that are widely distributed in the whole world, and recent studies suggest that *Aplosporella* species are not host-specific [31]. The 264 records of *Aplosporella* are listed in Index Fungorum [16], however, only a few species have sequence data in the GenBank database. In this study, *A*. *artocarpi* associated with mangoes was found in China, and the phylogenetic placements of *Aplosporella* taxa are shown in Figure 12.

**Figure 13:**
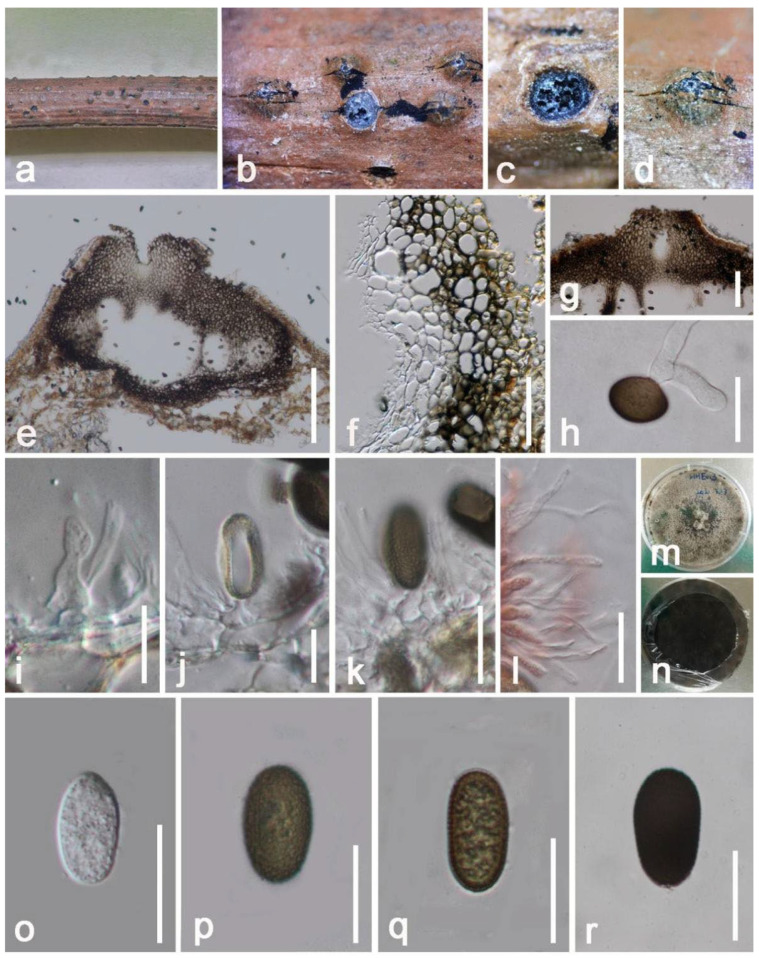
*Aplosporella artocarpi* (HKAS 122656). (**a**) Conidiomata on host surface; (**b**,**c**) Horizontal section of conidiomata; (**d**) Close-up of conidiomata; (**e**) Vertical section through conidiomata; (**f**) Peridium; (**g**) Ostiole; (**h**) Germinated conidium; (**i**–**k**) Conidia with conidiogenous cells; (**l**) Hamathecium stained by Congo red reagent; (**m**,**n**) Colony on PDA; (**o**–**r**) Immature to mature conidia. Scale bars: (**e**) = 200 μm; (**g**) = 100 μm; (**f**,**h**) = 20 μm; (**k**,**l**,**o**–**r**) = 15 μm; (**i**,**j**) = 10 μm.

*Aplosporella artocarpi* Trakun., L. Lombard & Crous, Persoonia 34: 91 (2014) (Figure 13)

Index Fungorum number: IF 810167

*Saprobic* on dead branch of *Mangifera indica*. Sexual morph: Undetermined. Asexual morph: *Conidiomata* 340–430 × 620–670 μm (x = 350 × 648 μm, *n* =10), erumpent to complete immersed from bark surface, discoid or irregular in shape, dark brown to black, multilocular (1–3-loculate), pore-like ostiolar at the centre, opened at mature, conspicuous. *Disc* 30–60 μm (x = 44 μm, *n* = 10) wide, black, circular. *Locules* quadrilateral to subglobose, or irregular in shape, 2–3 subdivided chambers were separated by vertical inner walls. *Ostioles* 150–210 × 220–280 μm μm (x = 180 × 245 μm, *n* = 10), single. *Pycnidial walls* 50–60 μm wide at the sides and bottom, 115–170 μm wide at the apex, unequal thickness, multilayer, outer layers composed of dark-brown cells of *textura globulosa*, becoming pale-brown and thin towards the inner areas, thick-walled, out-layer cells fusing with host cells. *Hamathecium* 1–2.5 μm wide (x = 2 μm, *n* = 15), cylindrical, raised among conidia, hyaline, smooth-walled, aseptate, moderately dense granules, paraphyses. *Conidiophores* reduced to conidiogenous cells. *Conidiogenous cells* 7–14 × 3–8 μm (x = 11× 6 μm, *n* = 15), holoblastic, hyaline, cylindrical to irregular, smooth-walled, without branching. *Conidia* 14–19 × 7–10 μm (x = 16 × 9 μm, *n* = 20), broadly ellipsoidal to subcylindrical, aseptate, initially hyaline and later become brown to dark brown, blunt ends, rough-walled, guttulate, verruculose.

Culture characteristics: Colonies on PDA 20 mm in diameter after one week at 25 °C in natural light, circular, flat to effuse, superficial, entire edge, white to gray when its young, hyphae green to brown at the centre, raised hyphal mass after one month, conidia formation in cultures after half year; dark brown to black at the reverse, without pigments produced in PDA. Vegetative hyphae 1-3 μm wide, hyaline, septate, and conidia are same to above descriptions.

Substrata: on twigs of *Artocarpus heterophyllus* [81]; on a dead branch of *Mangifera indica* (this study).

Distribution: Thailand [81]; China (this study).

Material examined: China, Yunnan Province, Honghe Menglong Village on a dead branch of *Mangifera indica,* (102°50′11″ E, 23°41′01″ N, 500 m), 22 December 2020, E.F. Yang, HHE013 (HKAS 122656), living culture KUMCC 21-0460. GenBank numbers: ITS: OL989220, LSU: OL989222, *tef1-α*: ON468651.

Notes: Our new strain (KUMCC 21-0460) clusters together with *A*. *artocarpi* (CPC 22791) and *A*. *chromolaenae* (MFLUCC 17-1517) with high statistical supports (99% in ML, 0.99 in BI) (Figure 12). It also shares similar morphological characteristics with the type specimen of *A*. *artocarpi* (CPC 22791) by conidiomata mostly solitary, semi-immersed to fully immersed, bursts out of the bark, and pore-like ostiole. In addition, when comparing conidiomata and conidia size of *A*. *artocarpi* (CPC 22791), our strain (KUMCC 21-0460), and *A*. *chromolaenae* (MFLUCC 17-1517) have similar size conidiomata (350–650 × 490–700 µm vs. 340–430 × 620–670 vs. 360–430 × 685–780 μm), and conidia (17–22 × 9–11 µm vs. 14–19 × 7–10 µm vs. 13–20 × 8–12 µm) [31,81]. However, conidiomata of *A*. *chromolaenae* totally differ by having superficial, coriaceous, and gregarious conidiomata without ostiolar. The BLASTn results of KUMCC 21-0460 showed ITS and *tef1-α* have high similarity with *A*. *artocarpi* (Table 1), as Li et al. [83] proposal establish a new taxon, their nucleotide difference has to more than 1.5%, apparently, our isolate does not meet this condition. Unfortunately, LSU of *A*. *artocarpi* (CPC 22791) and *tef1-α* of *A*. *chromolaenae* (MFLUCC 17-1517) were not available. Our phylogeny shows that *A*. *chromolaenae* (MFLUCC 17-1517) and *A*. *artocarpi* (CPC 22791, KUMCC 21-0460) cluster together (Figure 12). Thus, in the future, *tef-α* genes will be needed to resolve these two species, while the molecular data and morphological features confirmed that our collection is *A*. *artocarpi* and this is a new geographical and new host record of *A*. *artocarpi* on *Mangifera indica* from China (Table 2).

Sordariomycetes O.E. Erikss. & Winka, Myconet 1(1): 10 (1997)

Xylariales Nannf., Nova Acta R. Soc. Scient. upsal., Ser. 4 8(no. 2): 66 (1932)

Hypoxylaceae DC., in Lamarck & de Candolle, Fl. franç., Edn 3 (Paris) 2: 280 (1805)

*Hypomontagnella* Sir, L. Wendt & C. Lambert, Mycological Progress 18: 190 (2018)

Index Fungorum number: IF 827251

Type species: *Hypomontagnella monticulosa* (Mont.) Sir, L. Wendt & C. Lambert, Mycological Progress 18: 190 (2018)

Notes: *Hypomontagnella* (*Hy*.) was segregated from *Hypoxylon* by Lambert et al. [52], with *Hy. monticulosa* as the type species. Its basionym was *Hypoxylon monticulosum* that was collected from dead wood in France [89], after re-identified, *Hypomontagnella* accommodates six species, such as *Hy. austrobahiensis*, *Hy. barbarensis, Hy. monticulosa, Hy. rubigineoareolata* and *Hy. submonticulosa*. The sexual morph of *Hypomontagnella* taxa was described by spherical to obovoid perithecia, with slightly raised ostioles, minutely to conspicuous conical papillate surrounded by a black disc, eight-spored asci, stipitate, persistent, cylindrical, amyloid, and producing ellipsoid-inequilateral ascospores, with broadly or narrowly rounded ends, transversally striate or smooth [52]. The asexual morph of this genus is characterized by conidiophores with virgariella-like branches, rarely nodulisporium-like branches, conidia usually subglobose to fusiform, granular or verruculose [52]. According to Lambert et al. [52], it was mentioned that *Hypomontagnella* has perispores smooth or with transversally striate or namentations, and this is the key characteristic that differs the genera *Annulohypoxylon* and *Jackrogersella*. In addition, *Hypomontagnella* differs from *Hypoxylon* in having woody to carbonaceous ascomata. *Hypomontagnella* does not have reddish granules, ostiole central, conical papillate, usually with a black annulate disc, none detectable pigments are produced when soak in KOH solution. The new species *Hypomontagnella spongiphila* associated with marine habitat was introduced by Wibberg et al. [90], and the antibacterial and anticancer metabolites from *Hy. monticulosa* have been reported by Anisa et al. [91]. The phylogeny of *Hypomontagnella* and closely related genera is shown in Figure 3.

**Figure 14:**
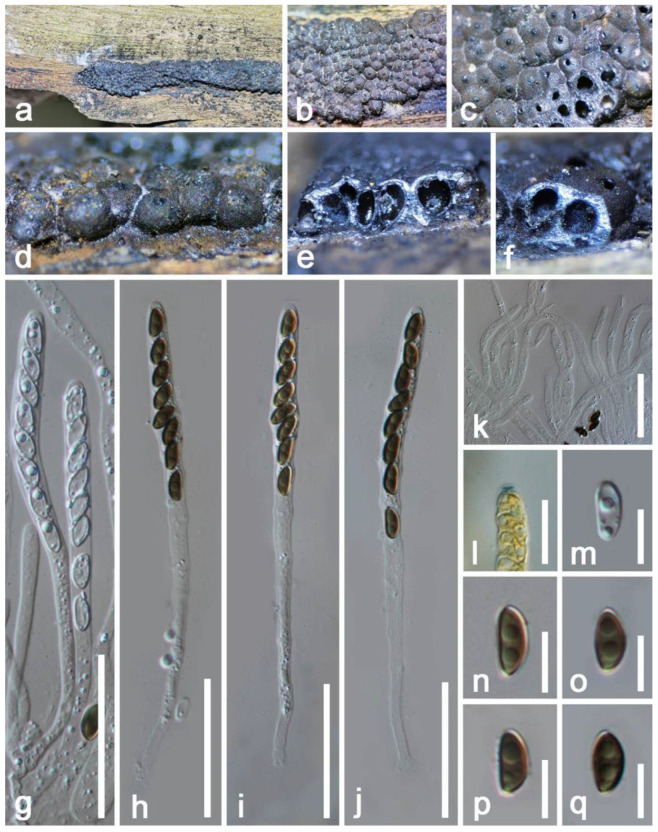
*Hypomontagnella monticulosa* (HKAS 122664). (**a**–**c**) Stromata in wood; (**d**) Stromatal surface with ostioles; (**e**,**f**) Cross section of stroma; (**g**–**j**) Asci; (**k**) Paraphyses; (**l**) “J+” apical apparatus. (**m**–**q**) Ascospores. Scale bars: (**g**–**k**) = 50 μm; (**l**–**q**) = 10 μm.

*Hypomontagnella monticulosa* (Mont.) Sir, L. Wendt & C. Lambert, Mycological Progress 18: 190 (2018) (Figure 14)

Index Fungorum number: IF 827252

*Saprobic* on dead branch of *Mangifera indica*. Sexual morph: *Stroma* effused-pulvinate, raised, conspicuous on host surface, superficial with a subiculum, reddish brown to blackish, globose, coriaceous, distinct ostioles central, conical papillate, woody to carbonaceous tissue, without KOH-extractable pigments. *Perithecia* spherical to obovoid, ostioles higher than the stromatal surface. *Hamathecium* 3.5–6 µm wide, hyaline, septate, unbranched, contains oil droplets, generate from a gelatinous matrix at the base, paraphyses. *Asci* 147–173 × 6.5–10 μm (x = 160 × 8 μm, *n* = 20), 8-spored, unitunicate, oblong to cylindrical, hyaline, apically rounded, with long pedicellate, with a “J+” apical apparatus. *Ascospores* 10–12 × 4–6 μm (x =11 × 5.5 μm, *n* = 20), uniseriate, fusiform to ellipsoid-inequilateral, hyaline, and turning brown to luteous-brown when mature, frequently narrowly rounded ends, without oblique or sigmoid germ slit, thick and smooth-walled, aseptate, with 1–2 oil droplets, perispore eventually dehiscent in 10% KOH, epispore smooth. Asexual morph: Undetermined.

Material examined: China, Yunnan Province, Honghe Menglong Village on a dead branch of *Mangifera indica*, (102°50′11″ E, 23°41′01″ N, 500 m), 22 December 2020, E.F. Yang, HHE004 (HKAS 122664). GenBank numbers: ITS: OL989326, LSU: OM001328, β-tubulin: ON468657, *rpb2*: ON392010.

Substrata: Unknown host species [92,93]; *Aleurites moluccanus* [94]; *Cladonia leporina* [95]; Lichen, Sargassum seaweed [96]; on decaying dicot wood [48]; *Platostoma palustre* [97]; endophytic fungal of fresh rhizome of *Zingiber griffifithii* [91]; branch of *Leucaena leucocephala* [98]; branch of *Mangifera indica* (this study).

Distribution: Thailand [93,98]; USA [94,95], Western Province, Sri Lanka [48]; China [92,97], this study); North Sumatra, Indonesia [91]. Indonesia, Malaysia [96].

Notes: Based on morphology, our isolate fits with the concept of *Hypomontagnella* which was described by Lambert et al. [52], and is characterized by ascomata effused-pulvinate, raised, reddish-brown to black, carbonaceous tissue, ostioles central, conspicuous, perithecial mounds, encircled usually by a superficial black disc, without colored granules and ascomatal pigments. Additionally, the BLASTn results (ITS, LSU, *rpb2* and β-tubulin) showed that our isolate belongs to *Hy. monticulosa* with high similarity (>99%) (Table 1). Multi-gene phylogenetic analyses (ML and BI) also showed HKAS (122664) clusters with *Hy. monticulosa* (CLL 205, MUCL54604) with high statistical supports (100 in ML, 0.99 in BI) (Figure 3). Therefore, our isolate is identified as *Hy. monticulosa* based on morphological examination and phylogenetic analyses.

Diatrypaceae Nitschke, Verh. Naturhist. Vereines Preuss. Rheinland: 73 (1869)

*Paraeutypella* L.S. Dissan., J.C. Kang, Wijayaw. & K.D. Hyde, Biodiversity Data Journal 9: e63864, 11 (2021)

Index Fungorum number: IF 557954

Type species: *Paraeutypella guizhouensis* L.S. Dissan., J.C. Kang & K.D. Hyde, Biodiversity Data Journal 9: e63864, 12 (2021)

Notes: The *Paraeutypella* (*P*.) is a recently established genus by Dissanayake et al. [26], and the generic type, *P*. *guizhouensis* was found saprobic on dead twigs from China. To date, three species were accepted in this genus (*P*. *citricola, P*. *guizhouensis* and *P*. *vitis*). *Paraeutypella citricola* and *P*. *vitis* were previously placed in *Eutypella*. The sexual morph of *Paraeutypella* is characterized by having immersed, erumpent, solitary or aggregated stromata, ascomata with groups of 4–25 perithecia, surrounded by white, powdery entostroma, elongated ostiolar neck, with elongate, filiform, narrow, unbranched, septate, guttulate paraphyses, 8-spored asci, with long pedicellate, clavate to cylindrical clavate or spindle-shaped, with a “J-” apical apparatus, ascospores allantoid, hyaline to light brown, sometimes yellow, biseriate, contain oil droplets. The sexual morph is described as coelomycetous, forming black, subconic, multiloculate, largely prosenchymatous conidiomata, conidiogenous cells cylindrical, proliferating percurrently or sympodially, produced with hyaline, single-celled, slightly to moderately curved conidia, and flattened bases, guttulate [26,99,100].

**Figure 15:**
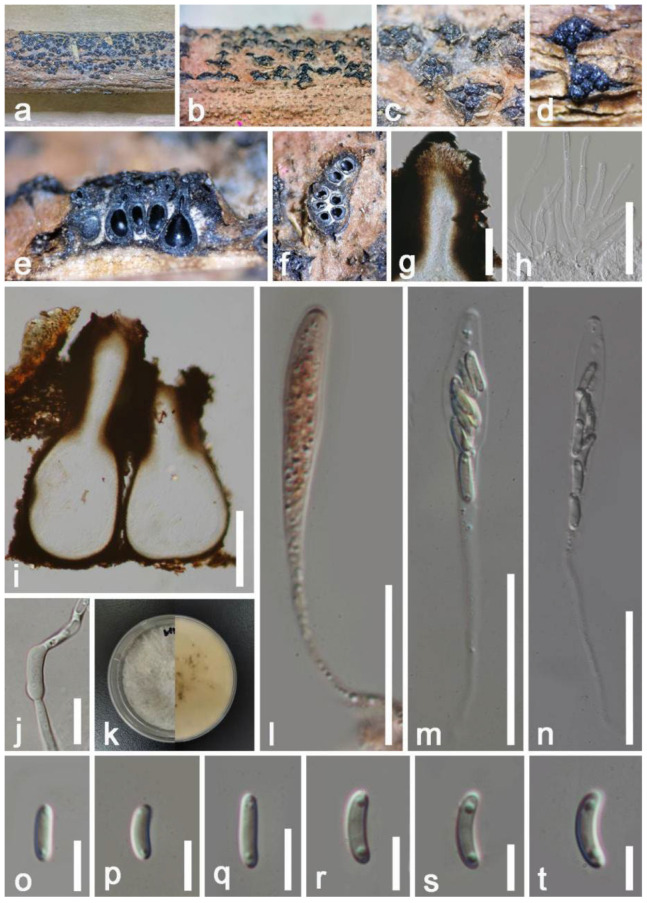
*Paraeutypella citricola* (HKAS 122667). (**a**–**d**) Stromatal tissue with perithecia on natural substrate; (**e**,**i**) Vertical sections of stromata; (**f**) Transverse sections of stromata; (**g**) Neck; (**h**) Paraphyses; (**j**) Germinated ascospore; (**k**) Colonies on PDA; (**l**) Immature ascus stained by Congo red reagent; (**m**,**n**) Asci; (**o**–**t**) Ascospores. Scale bars: (**i**) = 250 µm; (**g**) = 100 µm; (**h**) = 50 µm; (**m**,**n**) = 30 µm; (**l**) = 20 µm; (**j**,**o**–**t**) = 5 µm.

*Paraeutypella citricola* (Speg.) L.S. Dissan., Wijayaw., J.C. Kang & K.D. Hyde, Biodiversity Data Journal 9: e63864, 14 (2021) (Figure 15)

Index Fungorum number: IF 558003

*Saprobic* on dead branch of *Mangifera indica*. Sexual morph: *Stromata* semi-immersed, erumpent, aggregated, circular to irregular, carbonaceous. *Ascomata* perithecial, irregular, with groups of 6–12 perithecia arranged in a valsoid configuration, distinctly visible scattered black region on host surface and confluent into large groups. *Ascomata* (exclude neck) 343–424 µm × 240–340 µm (x = 384 × 293 μm, *n* = 10), subglobose to ovoid, black-brown, surrounded by white, powdery entostroma. *Ostioles* long ostiolar neck 215–390 × 138–190 µm (x = 302 × 164 μm, *n* = 10), cylindrical to subcylindrical, prolonged, emerging singly or in groups through the bark. *Peridium* 22–42 µm wide, multilayer, comprised of dark-brown cells of *textura prismatica* in the inner layers, hyaline cells of *textura prismatica* in the outter layers. *Hamathecium* 4–6 µm wide, subhyaline, widen at the base, septate, dense, slightly constricted at the septa, granulate, obtuse ends, embedded on a gelatinous matrix, paraphyses. *Asci* 61–95 × 4.5–6.5 µm (x = 78 × 5.5 μm, *n* = 45), 8-spored, unitunicate, cylindrical-clavate, straight to flexuous, long pedicellate, with a “J-” subapical apparatus. *Ascospores* 7.4–8.7 × 1.7–2.2 µm (x = 8 × 2 μm, *n* = 20), allaintoid to sub-allaintoid, subhyaline to yellowish, mostly curved at maturity, sometimes straight when young, smooth-walled, with two oil droplets, guttulate. Asexual morph: Undetermined.

Culture characteristics: Colonies on PDA 40 mm in diameter after two weeks at 27 °C. Colonies white, flat, cottony, with a fimbriate edge, with a medium density; grayish at the reverse, without pigments produced in PDA. Vegetative hyphae 1-3 μm wide, hyaline, septated, branched.

Substrata: *Citrus* sp. [101,102,103]; *Melia azedarach* [44]; *Vitis* sp. [104]; *Salix* sp. [105]; *Pistacia vera* [106]; *Acer palmatum* (Sapindaceae) [26]; a dead branch of *Mangifera indica* (this study).

Distribution: Philippines [102]; Australia [103]; South Africa [44]; United States [104]; Iran [105,106]; China ([26,101], this study).

Material examined: China, Yunnan Province, Honghe Menglong Village, on a dead branch of *Mangifera indica*, (102°50′11″ E, 23°41′01″ N, 500 m), 22 December 2020, E.F. Yang, HHE0121, (HKAS 122667), living culture KUMCC 21-0461. GenBank numbers: ITS: OL989101, LSU: OL989150, β-tubulin: ON468663.

Notes: The morphological comparison of *P. citricola* IRAN2349C (basionym: *Eutypella citricola*) with our collection KUMCC 21-0461 showed they both have erumpent, scattered stromata, non-prominent ostioles, long neck, pustules with sulcate perithecial beaks, almost same size in asci (50–80 (–90) × 6–8 µm vs. 61–95 × 4.9–6.3 µm) long pedicellate, and allantoid, hyaline or yellowish ascospores (7–11(–12) × 1.7–2.3 µm vs. 7.4–8.7 × 1.7–2.2 µm), with distinct oil droplets at each ends when mature [46,107]. In addition, the BLASTn results of ITS, LSU, and β-tubulin showed high similarity (>99%) with *P*. *citricola* strains (Table 1) and the multi-gene phylogenetic analyses based on ITS and β-tubulin also showed moderate statistical supports in ML, and clustered well with *P*. *citricola* clade (HUEFS 194248, IRAN 2349C, CBS 128330) (Figure 1). Therefore, we report our collection as a new host record of *P*. *citricola* from a dead branch of *Mangifera indica*.

Calosphaeriales M.E. Barr, Mycologia 75(1): 11 (1983)

Pleurostomataceae Réblová, L. Mostert, W. Gams & Crous, Stud. Mycol. 50(2): 540 (2004)

*Pleurostoma* Tul. & C. Tul., Selecta Fungorum Carpologia, Tomus Secundus. Xylariei-Valsei-Sphaeriei 2: 247 (1863)

Index Fungorum number: IF 4247

Type species: *Pleurostoma candollei* Tul. & C. Tul., Selecta Fungorum Carpologia, Tomus Secundus. Xylariei-Valsei-Sphaeriei 2: 247 (1863)

Notes: *Pleurostoma* (*Pl*.) was established by Tulasne & Tulasne [108] with *Pl. candollei* as the type species. It currently accommodates a total of five species in Index Fungorum [16], and most species have molecular data in GenBank (2022) (https://www.ncbi.nlm.nih.gov/nuccore/, accessed on 4 May 2022). Two *Pleurostoma* (*Pl. vibratile* and *Pl. minimum*) have been reidentified based on phylogenetic analyses and transferred to *Phaeoacremonium* (Togniniaceae) [109]. Sexual morph of *Pleurostoma* is characterized by having semi-immersed to erumpent, superficial ascomata, *hamathecium* absent, obovoid, club-like, pedicellate asci, ascospore numerous, allantoid, aseptate, hyaline, extremely flexuous [110,111]. In addition, the asexual morphologies were mentioned by Tsang et al. and Huang et al. [32,112] as hyphomycetous, with hyaline to brown, branched, septate mycelia, projection-like conidiophores, phialides monophialidic or polyphialidic, cylindrical, erect, straight to fexuouse, hyaline to brown usually laterally located, clustered, having produced hyaline, ovoid to suballantoid, aseptate, smooth-walled conidia on the apex of the phialides, present as a slimy mass. In nature, *Pleurostoma* is widely distributed in woods, soil, and sewage worldwide, and was mostly reported in Iran, Spain, and Sri Lanka [8]. In addition, Tsang et al. [32] first reported one human-infected case associated with a dematiaceous fungus *Pl. hongkongense,* which was isolated from the subhepatic abscess pus and drain fluids of a patient. The phylogenetic relationships among the taxa in this genus were well-studied by Tsang et al. [32], and the updated phylogenetic tree of this study is shown in Figure 16.

**Figure 17:**
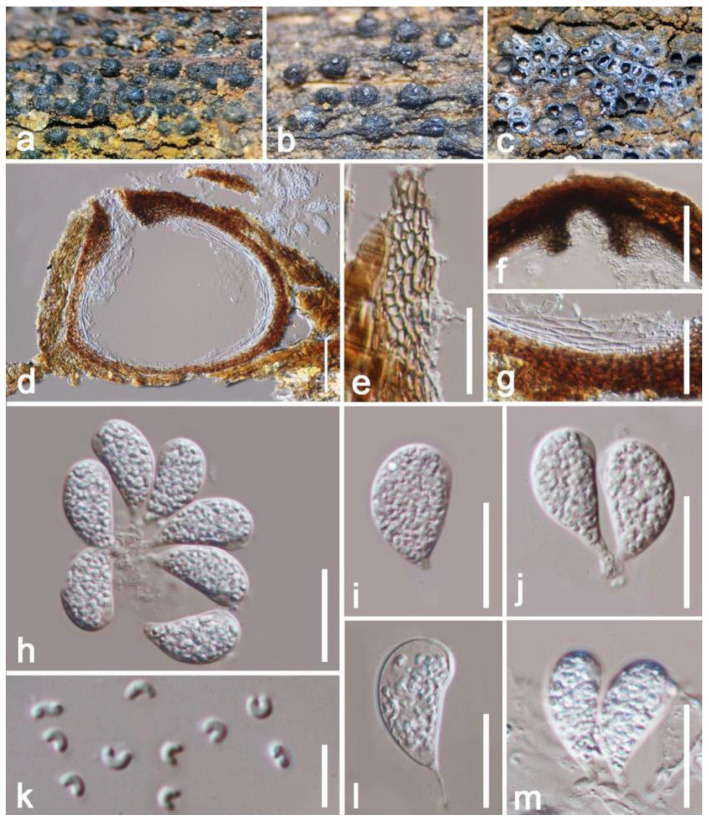
*Pleurostoma ootheca* (HKAS 122679) (**a**–**c**) Ascomata perithecial on dead wood of *Mangifera indica*; (**d**) Vertical section of ascomata; (**e**). Peridium at side; (**f**) Ostiole; (**g**) Peridium at base; (**h**–**j**,**l**,**m**) Asci; (**k**) Ascospores. Scale bars: (**d**,**e**) = 50 μm; (**g**) = 30 μm, (**h**–**j**,**l**,**m**) = 20 μm; (**k**) = 5 μm.

*Pleurostoma ootheca* (Berk. & M.A. Curtis) M.E. Barr, Mycologia 77: 564 (1985) (Figure 17)

Index Fungorum number: IF 105702

*Saprobic* on dead wood of *Mangifera indica*. Sexual morph: *Ascomata* perithecial 169–244 × 189–310 μm (x = 207 × 250 μm, *n* = 20), subglobose to ampulliform, solitary to gregarious, semi-immersed to erumpent, black, conspicuous at the surface, coriaceous, shiny, carbonaceous, ostiolar, without papillate. *Ostioles canal* 40–55 × 50–65 μm (x = 47 × 57 μm, *n* = 20), narrowly, mostly in central, brown to black. *Peridium* 12–16 μm wide, thin-walled, unequal in thickness, thicken near the neck, muti-layers, comprised of compressed *textura prismatica,* hyaline cells at the inner layers 8–13 μm, membranaceous, and composed of pale brown to dark brown cells of *textura intricata* to *textura epidermoidea* at the middle layers. *Hamathecium* not observed. *Asci* 23–28 × 10–14 μm (x = 26 × 12 μm, *n* = 20), multi-spored, unitunicate, obovoid with straight or slightly curved at the apical, hyaline, thick-walled, apically rounded, ocular chamber absent, club-like and short pedicellate. *Ascospores* 3–5 × 1–2 μm (x = 4.5 × 1.5 μm, *n* = 30), numerous, allantoid, aseptate, smooth-walled, hyaline, extremely flexuous, obtuse ends, defined outline, scattered irregularly in the asci. Asexual morph: Undetermined

Substrata: *Quercus agrifolia* [113]; dead wood of *Mangifera indica* (this study).

Distribution: California, USA [113]; Yunnan, China (this study).

Material examined: China, Yunnan Province, Honghe Menglong Village on dead wood of *Mangifera indica*, (102°50′11″ E, 23°41′01″ N, 500 m), 22 December 2020, E.F. Yang, HHE025 (HKAS 122679). GenBank numbers: ITS: OM017217, LSU: OM017219, SSU: OM017218, β-tubulin: ON468660, *rpb2*: ON392011.

Notes: The fungus (HKAS 122679) shares similar characteristics with the type species *Pl. ootheca* which was described by Barr [114], and fits with *Pl. ootheca* (CBS 115329) [110,111]. The BLASTn results of four genes (ITS, LSU, SSU, and β-tubulin) showed high similarities with *Pl. ootheca* (Table 1). Moreover, the phylogenetic trees from BI and ML also showed our isolate (HKAS 122679) clusters well with *Pl. ootheca* (CBS 115329) with high statistical support (100% in ML and 1.00 in BI) (Figure 16). Therefore, our strain as *Pl. ootheca* and also this is a new host and country record from *Mangifera indica* in China.

## 4. Discussion

**Figure 1:**
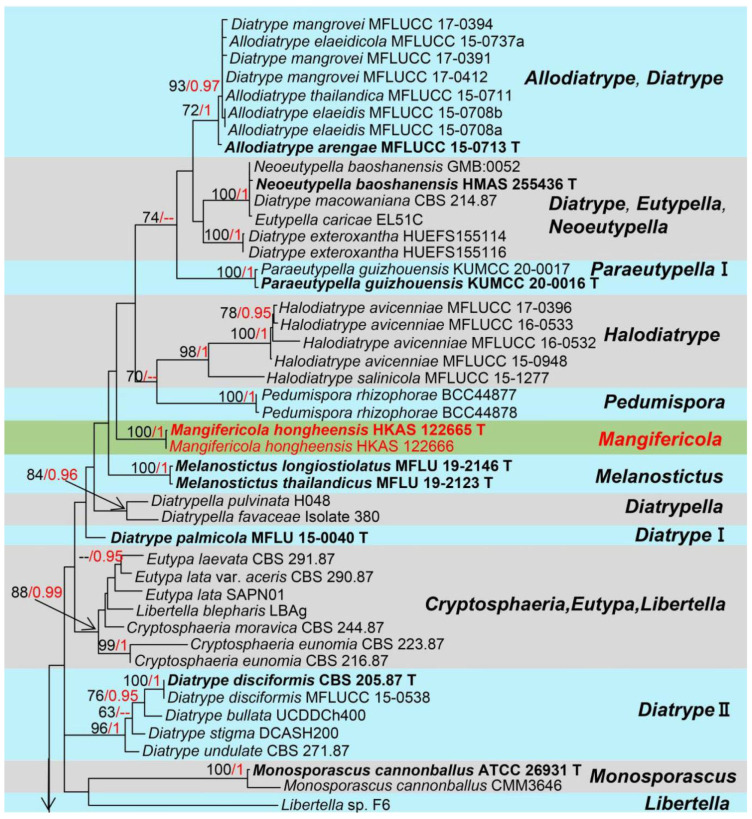

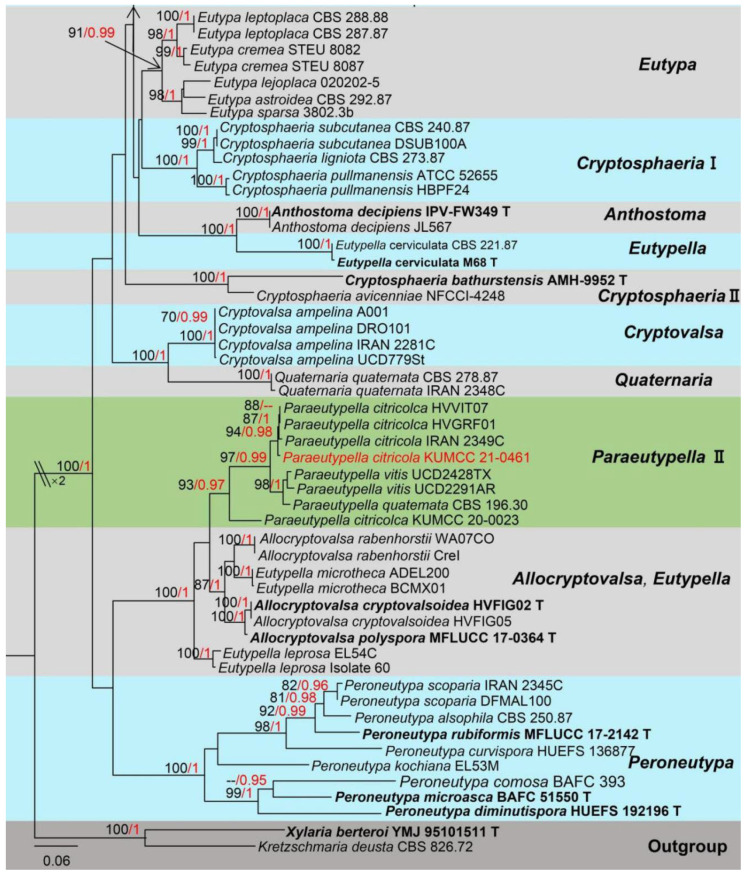
Phylogram of a novel genus *Mangifericola,* a new host record of *Paraeutypella citricola*, and other genera within the family Diatrypaceae generated from maximum likelihood analysis based on a combined ITS, β-tubulin sequence datasets, with *Xylaria berteroi* (YMJ 95101511) and *Kretzschmaria deusta* (CBS 826.72) as the outgroups. Related sequences used in the phylogeny were taken from Dissanayake et al. [26]. The species introduced in this study are indicated in red, and the type strains are indicated in bold with “T”. Bootstrap values equal to or greater than 70% (ML, Left) and Bayesian posterior probabilities (BI, right) equal to or greater than 0.95 are given at the nodes. Hyphens (-) represent values less than 70% in ML/0.95 in BI. For more information, please see Supplementary Materials (Table S1, Supplementary Information S1).

**Figure 2:**
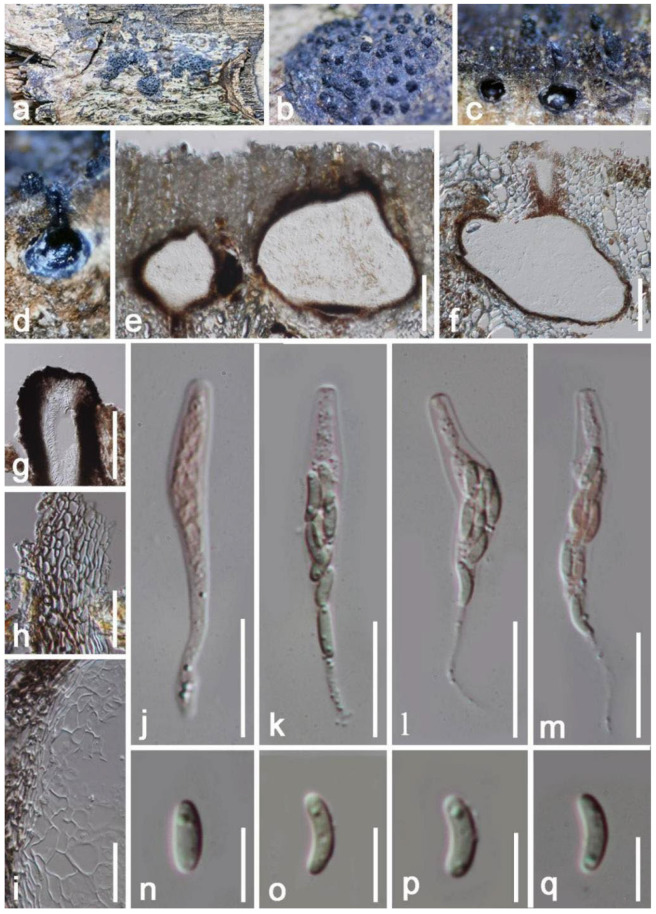
*Mangifericola hongheensis* (HKAS 122665, holotype). (**a**,**b**) Appearance of stromatal tissue with perithecia; (**c**–**f**) Vertical sections through stromata; (**g**) Section through the neck; (**h**) Peridium at outer layers; (**i**) Peridium at inner layers; (**j**–**m**) Asci (stained with Congo red); (**n**–**q**) Ascospores. Scale bars: (**e**,**g**) = 100 µm; (**f**) = 50 µm; (**i**,**h**) = 20 µm; (**j**–**m**) = 15 µm; (**n**–**q**) = 5 µm.

**Figure 3:**
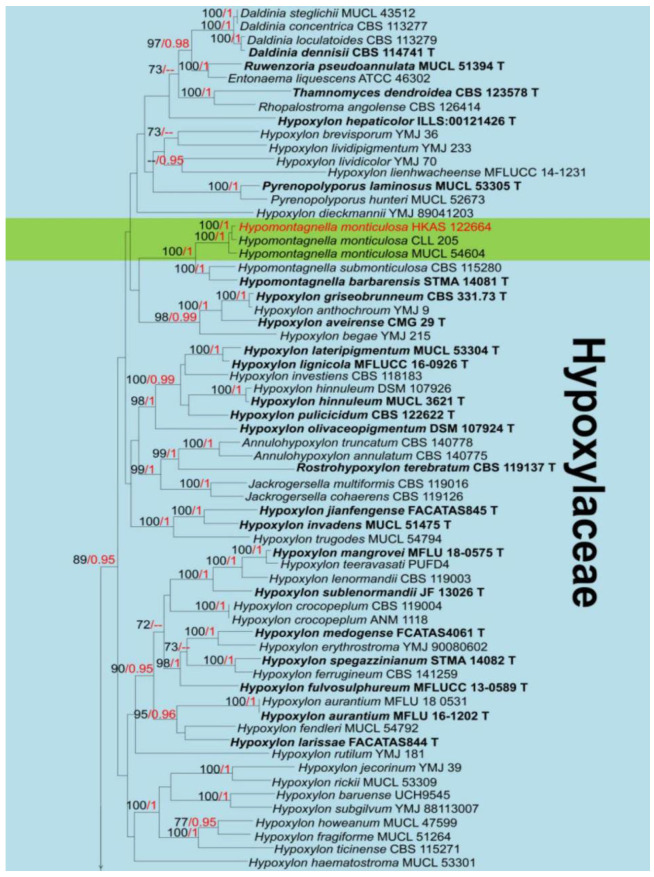

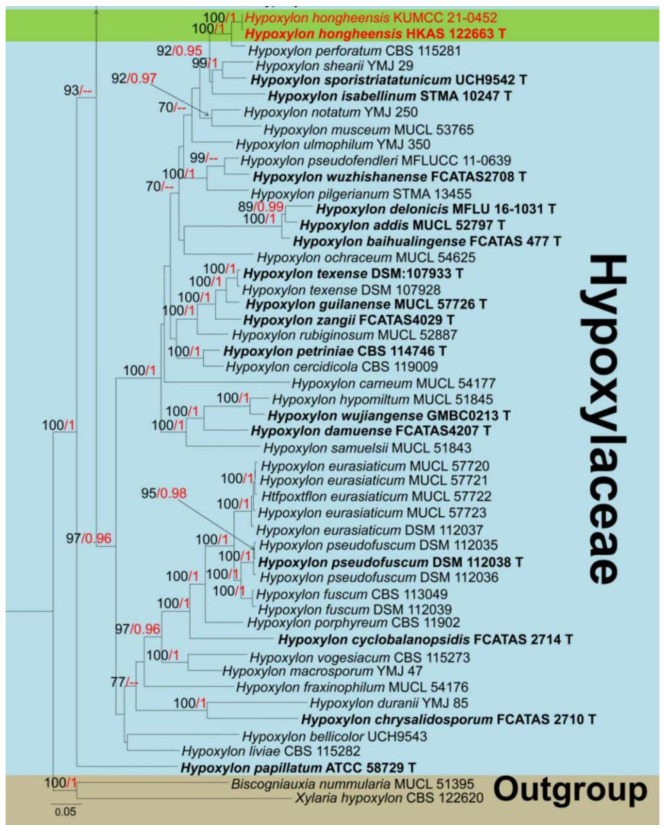
Phylogram of a new species *Hypoxylon hongheensis*, a new host record of *Hypomontagnella monticulosa,* and related genera within the family Hypoxylaceae generated from Maximum Likelihood analysis based on a combined LSU, β-tubulin, ITS and *rpb2* sequence datasets, with *Biscogniauxia nummularia* (MUCL 51395) and *Xylaria hypoxylon* (CBS 122620) as the outgroups. Related sequences used in the phylogeny were taken from Song et al. [29]. The species introduced in this study are indicated in red, and the type strains are indicated in bold with “T”. Bootstrap values equal to or greater than 70% (ML, Left) and Bayesian posterior probabilities (BI, right) equal to or greater than 0.95 are given at the nodes. Hyphens (-) represent values less than 70% in ML/0.95 in BI. For more information, please see Supplementary Materials (Table S2, Supplementary Information S2).

To date, around 160 records of mango-associated xylarialean fungi have been documented in the U.S. National Fungus Collections Fungal Database [16]. This study, a novel genus *Mangifericola* (Diatrypaceae) is established with *M*. *hongheensis* as the type species. Based on the BLASTn results of ITS, and LSU, our new genus *Mangifericola* is closely related to *Diatrype* and *Diatrypella,* but the BLASTn result of *tub2* indicated that it is a distinct genus (Table 1). In addition, *Mangifericola* has the characteristics of having ascomata groups fully immersed with a long and erect neck, and the colony characteristics are similar to the *Diatrype palmicola* (MFLU 15-0040) [46], but they are distinguished by ITS, *tub2* gene regions and phylogenetic analyses (Figure 1). The new host record of *Paraeutypella citricola* (Diatrypaceae) is also introduced in this study based on morphology and multigene phylogeny. The sizes, asci, and ascospore morphology of our strain well matched with previous studies of *P*. *citricola* [46,107], and the BLASTn results of ITS, LSU, and *tub2* also showed a high similarity (>99%) with *P*. *citricola* (Table 1). The BLASTn results of *Hypoxylon hongheensis* (Hypoxylaceae) indicated that it is closely related to sister species *H. perforatum,* however, they differ due to the base pair differences of ITS, *rpb2, tub2* (Table 1), asci size and ascomatal pigments in 10% KOH solution [55,56]. Another new host record of a xylarialean fungus, *Hypomontagnella monticulosa* (Hypoxylaceae) is also described here based on morphology and multigene phylogeny (Figure 3).

Many species in the Botryosphaeriales have been reported on mango, of which two species belong to the Aplosporellaceae, while 60 species belong to the Botryosphaeriaceae [16]. *Aplosporella artocarpi* (Aplosporellaceae) is introduced as a new host and country record in this study. As the LSU of *A*. *artocarpi* (CPC 22791) and *tef1-α* of *A*. *chromolaenae* (MFLUCC 17-1517) were not available, thus morphological comparisons (Table 2) are provided to support our isolate is *Aplosporella artocarpi*. We also report two other new collections of *Lasiodiplodia theobromae* and *L. pseudotheobromae* (Botryosphaeriaceae), and they all have previously been isolated from mango in China [78,79,85]. The mature conidia of *Lasiodiplodia pseudotheobromae* (HKAS 122658) and *L. theobromae* (HKAS 122660) were not found, but the BLASTn results, conidia morphology, and phylogenetic analyses result fully supported the identification.

**Figure 5:**
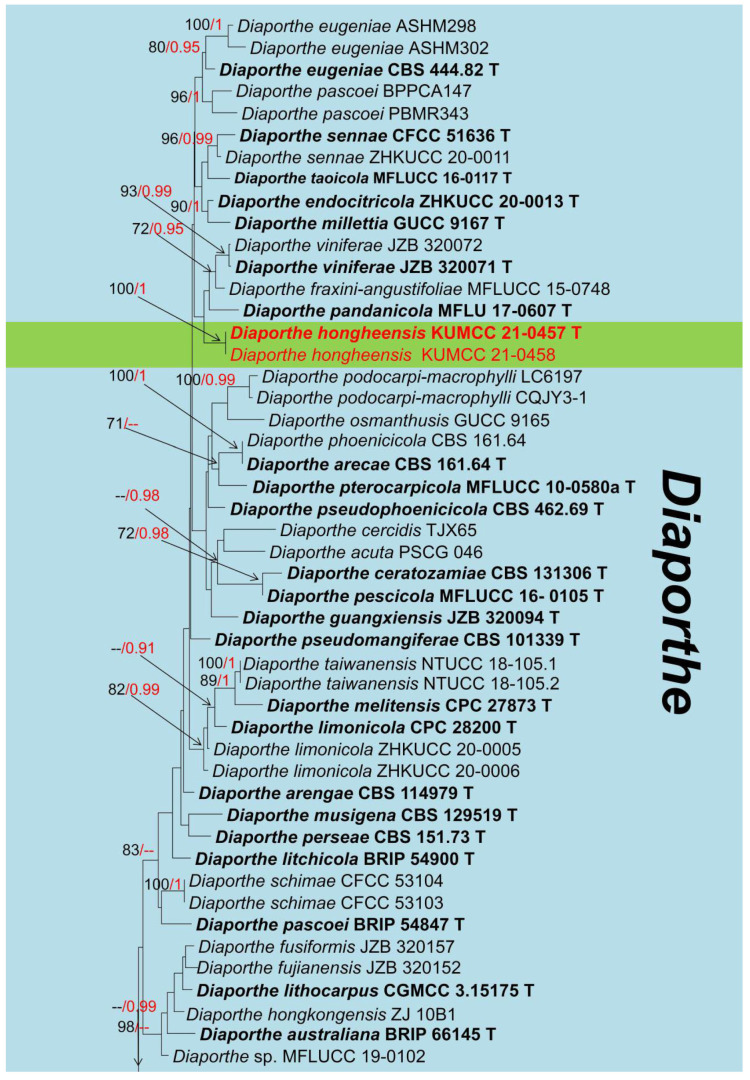

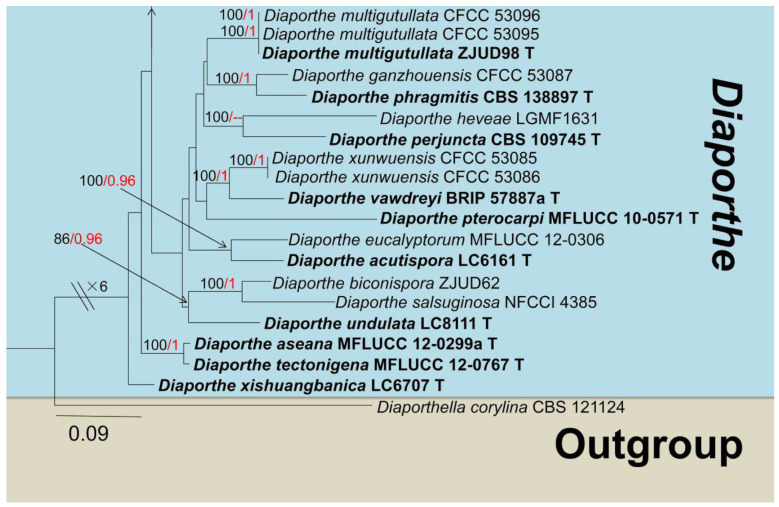
Phylogram of a new species *Diaporthe hongheensis* and closed species within genus *Diaporthe* generated from maximum likelihood analysis based on a combined ITS, *tef1-α*, β-tubulin and CAL sequence datasets, with *Diaporthella corylina* (CBS 121124) as the outgroup. Related sequences used in the phylogeny were taken from Ariyawansa et al. [66] and Dong et al. [28]. The species introduced in this study are indicated in red, and the type strains are indicated in bold with “T”. Bootstrap values equal to or greater than 70% (ML, Left) and Bayesian posterior probabilities (BI, right) equal to or greater than 0.95 are given at the nodes. Hyphens (-) represent values less than 70% in ML/0.95 in BI. For more information, please see the Supplementary Materials (Table S3, Supplementary Information S3).

**Figure 12:**
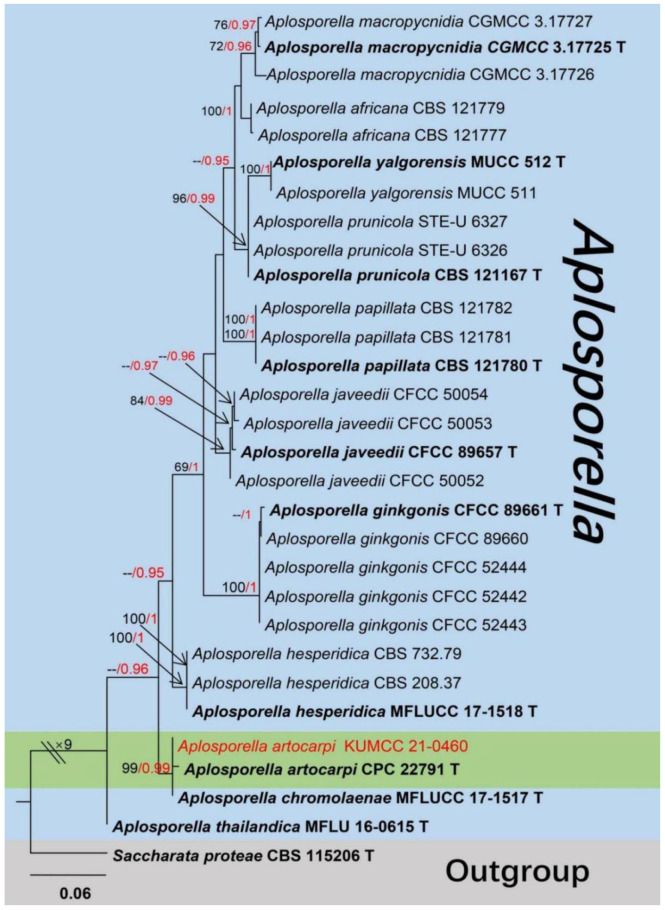
Phylogram of a new country and host of *Aplosporella artocarpi* and other species in genus *Aplosporella* generated from maximum likelihood analysis based on a combined LSU, ITS and *tef1-α* sequence datasets, with *Saccharata proteae* (CBS 115206) as the outgroup. Related sequences used in the phylogeny were taken from Mapook et al. [31]. The species introduced in this study are indicated in red, and the type strains are indicated in bold with “T”. Bootstrap values equal to or greater than 70% (ML, Left) and Bayesian posterior probabilities (BI, right) equal to or greater than 0.95 are given at the nodes. Hyphens (-) represent values less than 70% in ML/0.95 in BI. For more information, please see the Supplementary Materials (Table S6, Supplementary information S6).

**Figure 16:**
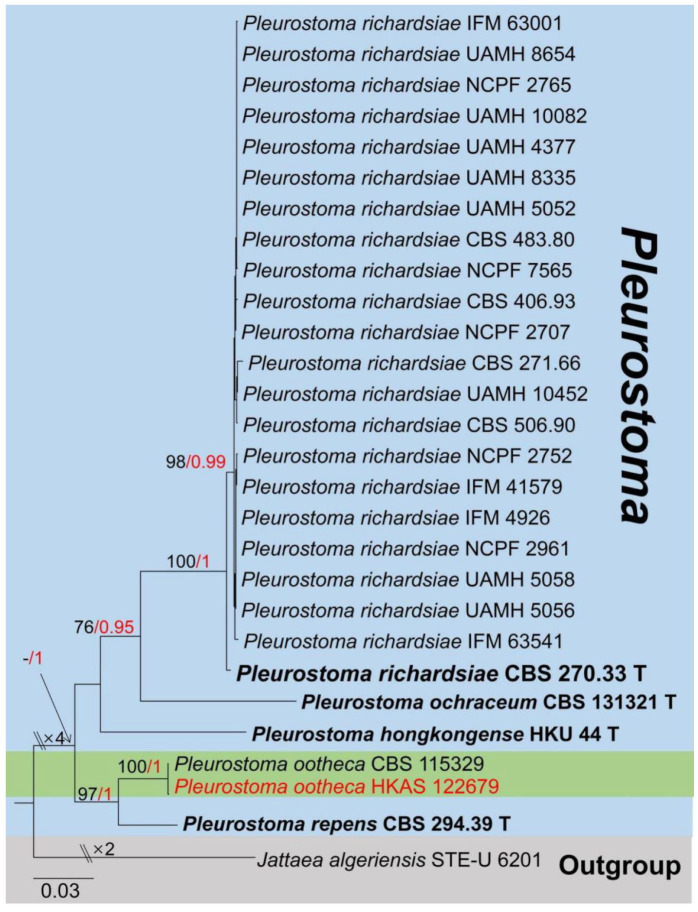
Phylogram of a new country and host record of *Pleurostoma ootheca* and other species in the genus *Pleurostoma* generated from maximum likelihood analysis based on a combined LSU, SSU, ITS, β-tubulin sequence datasets, with *Jattaea algeriensis* (STE-U 6201) as the outgroup. Related sequences used in the phylogeny were taken from Tsang et al. [32]. The species introduced in this study are indicated in red, and the type strains are indicated in bold with “T”. Bootstrap values equal to or greater than 70% (ML, Left) and Bayesian posterior probabilities (BI, right) equal to or greater than 0.95 are given at the nodes. Hyphens (-) represent values less than 70% in ML/0.95 in BI. For more information, please see the Supplementary Materials (Table S7, Supplementary information S7).

From the order Calosphaeriales, only *Calosphaeria mangiferae* (Calosphaeriaceae) has earlier been reported on *Mangifera indica* [16]. The *Pleurostoma* (Pleurostomataceae) are widely distributed in woods, soil, and sewage worldwide, and in addition, a dematiaceous fungus *Pl. hongkongense* was isolated from a patient [32]. In total, 73 records of mango-associated diaporthalesan fungi have been documented in the U.S. National Fungus Collections Fungal Database, of which 78% of the records (57 records) belong to *Diaporthe* and *Phomopsis* (Diaporthaceae) [16]. *Diaporthe* species reported were plant pathogens, saprobes, endophytes, or associated with humans and other mammals [78,87,88]. Our isolate *Diaporthe hongheensis* (HKAS 122657) is highly matched to the concept of sexual *Diaporthe* [29,78,84], and based on the BLASTn results and phylogenetic analyses support, we identified it as a new species (Table 1, Figure 5). A chaetothyrialean fungus viz. *Cyphellophora hongheensis* is reported associated with mango for the first time. Species of *Cyphellophora* (Cyphellophoraceae, Chaetothyriale) are epiphytic on excretions of insects or foliar epiphytes on living leaves, and some species are associated with human and animal skin and nails, including *Cyphellophora laciniata*, *C. europaea*, and *C. pluriseptata* [71,100]. In this study, our saprobic fungal isolate *C. hongheensis* was isolated from a living mango branch hanging on a mango tree and is characterized by ascomata that are superficial, and the absence of distinct pathogenetic symptoms.

In this study, we isolated four species of xylarialean fungi, three species of botryosphaerialen fungi, and three species from each order of Calosphaeriales, Chaetothyriales and Diaporthales. Based on the reports of the previous studies and this study, the species of Xylariales and Botryosphaeriales indeed have a high association with mango. Moreover, *Cyphellophora, Diaporthe* and *Pleurostoma* seem to have a wide range of adaptions for different hosts viz. plants, human and animals.

## Figures and Tables

**Table 1:** The BLASTn results of isolates from this study.

Taxa Names	Loci	The BLASTn Results
***Aplosporellaartocarpi*** (KUMCC 21-0460)	LSU(LR0R/LR5):	100% (571/571 bp; 0 gap) similar to *Aplosporella chromolaenae* (MFLUCC 17-1517)
	ITS(ITS4/ITS5):	100% (554/554 bp; 0 gap) similar to *Aplosporella artocarpi* (CPC 22791) and *A*. *chromolaenae* (MFLUCC 17-1517)
	*tef1-α*(688F/1251R):	100% (250/250 bp; 0 gap) similar to *Aplosporella artocarpi* (CPC 22791)
***Cyphellophorahongheensis*** (HKAS 122661)	SSU(NS1/NS4):	99% (964/966 bp; 0 gap) similar to *Cyphellophora sessilis* (CBS 238.93, CBS 243.85)
	LSU(LR0R/LR5):	98% (876/893 bp; 5 gaps) similar to *Cyphellophora sessilis* (SP12386Ca)
	ITS(ITS4/ITS5):	92% (509/556 bp; 8 gaps) similar to *Cyphellophora livistonae* (PhliHN2702, CPC 19433)
	*rpb1*(AR/CF):	75% (495/660 bp; 21 gaps) similar to *Cyphellophora oxyspora* (CBS 416.89, CBS 698.73)
***Diaporthehongheensis*** (KUMCC 21-0457)	ITS(ITS4/ITS5):	99% (556/562 bp; 2 gaps) similar to *Diaporthe eugeniae* (ASHM304) and *D*. *phaseolorum* (B3147)
	*tef1-α*(728F/986R):	96% (330/342 bp; 3 gaps) similar to *Diaporthe pascoei* (PBMR343)
	β-tubulin (Bt2a/Bt 2b):	100% (495/496 bp; 1 gap) similar to *Diaporthe endocitricola* (ZHKUCC 20-0013)
	CAL (228F/373R):	97% (468/480 bp; 3 gaps) similar to *Diaporthe* sp. (CFCC 53101)
***Hypomontagnellmonticulosa*** (HKAS 122664)	LSU(LR0R/LR5):	100% (841/842 bp; 0 gap) similar to *Hypomontagnella monticulosa* (MFLUCC 18-0362, XY02480)
	ITS(ITS4/ITS5):	99% (571/576 bp; 1 gap) similar to *Hypomontagnella monticulosa* (67.3.3)
	β-tubulin (Bt2a/Bt 2b):	97% (392/403 bp; 1 gap) similar to *Hypomontagnella monticulosa* (EK13014, YMJ 90080806)
	*rpb2*(5F/7cR):	99% (1074/1077 bp; 1 gap) similar to *Hypomontagnella monticulosa* (CLL 205)
***Hypoxylonhongheensis*** (HKAS 122663)	LSU(LR0R/LR5):	100% (840/841 bp; 1 gap) similar to *Hypoxylon perforatum* (XZ142)
	ITS(ITS4/ITS5):	98% (625/637 bp; 11 gaps) similar to *Hypoxylon perforatum* (MoEF023, KoRLI047347)
	β-tubulin(T1/T22):	97% (1386/1424 bp; 5 gaps) similar to *Hypoxylon perforatum* (STMA 14051) and *H. rubiginosum* (YMJ 4)
	*rpb2*(5F/7cR):	98 % (973/993 bp; 0 gap) similar to *Hypoxylon perforatum* (DSM:107930) and *H. rubiginosum* (FL1170)
***Lasiodiplodiatheobromae*** (HKAS 122660)	LSU(LR0R/LR5):	100% (846/847 bp; 0 gap) similar to *Lasiodiplodia krabiensis* (MFLU 17-2617) and *L*. *theobromae* (CBS 129758)
	ITS(ITS4/ITS5):	100% (498/498 bp; 0 gap) similar to *Lasiodiplodia theobromae* (ZW 50-1, IRNBS73)
	*tef1-α*(668F/986R):	100% (315/315 bp; 0 gap) similar to *Lasiodiplodia theobromae* (ZWLT 482, BOM230)
	*rpb2*(LasF/LasR):	100% (570/570 bp; 0 gap) similar to *Lasiodiplodia theobromae* (DAR82930)
	β-tubulin(Bt2a/Bt 2b):	99% (464/469 bp; 2 gaps) similar to Lasiodiplodia theobromae (LTHEOB 7940)
***Lasiodiplodiapseudotheobromae*** (HKAS 122658)	LSU(LR0R/LR5):	100% (862/862 bp; 0 gap) similar to *Lasiodiplodia hyalina* (CGMCC 3.17975) and *L. pseudotheobromae* (CBS 447.62)
	ITS(ITS4/ITS5):	100% (498/498; 0 gap) similar to *Lasiodiplodia pseudotheobromae* (AY-11, KoRLI047143)
	*tef1-α*(668F/986R:	100% (307/307 bp; 0 gap) similar to *Lasiodiplodia pseudotheobromae* (MPMR65, MFLUCC 17-2289, ZK201)
	*rpb2*(LasF/LasR):	100% (569/569 bp; 0gap) similar to *Lasiodiplodia pseudotheobromae* (DAR83095, CERC 3496)
	β-tubulin(Bt2a/Bt 2b):	100% (429/429 bp; 0 gap) similar to *Lasiodiplodia pseudotheobromae* (G32)
***Mangifericolahongheensis*** (HKAS 122665)	LSU(LR0R/LR5):	99% (844/856bp; 1 gap) similar to *Diatrype dalbergiae* (CBS 147068)
	ITS(ITS4/ITS5):	99% (552/557bp; 2 gaps) similar to *Diatrypella pulvinata* (B1B085-3-EM2CC568)
	β-tubulin(Bt2a/Bt2b):	90% (352/393bp; 18 gaps) similar to *Melanostictus thailandicus* (MFLU 19-2123, MFLU 19-2146)
***Paraeutypellacitricola*** (HKAS 122667)	LSU(LR0R/LR5):	100% (841/841 bp; 1 gap) similar to *Paraeutypella citricola* (CBS 128334, KUMCC 21-0461)
	ITS(ITS4/ITS5):	100% (531/531 bp; 0 gap) similar to *Paraeutypella citricola* (STEU 8186, KUMCC 21-0461, BRPET19)
	β-tubulin(Bt2a/Bt2b):	100% (370/373 bp; 1 gap) similar to *Paraeutypella citricola* (HUEFS 194248, HUEFS 131041)
***Pleurostoma ootheca*** (HKAS 122679)	SSU(NS1/NS4):	100% (1022/1022 bp; 0 gap) similar to *Pleurostoma ootheca* (CBS 115329) and *P. repens* (CBS 294.39)
	LSU(LR0R/LR5):	100% (559/560 bp; 0 gap) similar to *Pleurostoma ootheca* (CBS:115329, CMU 23858)
	ITS(ITS4/ITS5):	100% (519/519 bp; 0 gap) similar to *Pleurostoma ootheca* (CBS 126089) and *P. ootheca* (CBS 115329)
	*rpb2*(5F/7cR):	100% (940/947 bp; 4 gaps) similar to *Pleurostoma ootheca* (CBS 115329)
	β-tubulin(Bt2a/Bt 2b):	100% (451/451 bp; 0 gap) similar to *Pleurostoma ootheca* (CBS 115329)

**Table 2:** The morphology comparison of *A. artocarpi* and *A*. *chromolaenae*.

Species	Conidiomata	Conidia	References
***A.****artocarpi* (HKAS 122656)	340–430 × 620–670 μm, erumpent to complete immersed, discoid or irregular in shape, dark brown to black, multilocular, with a pore-like ostiolar.	14–19 × 7–10 μm, hyaline to dark brown, broadly ellipsoidal to subcylindrical, aseptate, blunt ends, rough-walled, guttulate.	This study
***A.****artocarpi* (CBS H-21931)	(350–)540–550(–650) × (490–)540–600(–700) µm, pycnidial, semi-immersed, mostly solitary, dark brown to black, with globose base, multilocular.	(17–)18–21(–22) × (9–)10–11 µm, ellipsoid to ovoid, smooth, moderately thick-walled, with granular content, aseptate.	[81]
***A.****chromolaenae* (MFLU 20-0298)	75–145 × 80–160 µm, gregarious, with 2–3 locules forming groups immersed in conidiostroma, globose to subglobose.	(13–)16–20 × 8.5–12 µm, hyaline to brown to dark brown, aseptate, ellipsoid or oval to reniform, thick-walled, verruculose.	[31]

## Data Availability

Not applicable.

## Supplementary Materials

The following supporting information can be downloaded at: https://www.mdpi.com/article/10.3390/jof8121249/s1, Tables S1–S7: The names, isolate numbers, and corresponding GenBank accession numbers of the taxa used in phylogenetic trees; Supplementary Information (S1–S7): the detail information of phylogenetic trees.
