# Supplementary material for: Taxonomy and Phylogeny of Fungi Associated with Mangifera indica from Yunnan, China
Source: J Fungi (Basel). 2022 Nov 26;8(12):1249. doi: 10.3390/jof8121249 (PMC9780836; doi:10.3390/jof8121249)
Supplement: Supplementary file 1 [file jof-08-01249-s001.zip › jof-1994577-supplementary.pdf]

# Supplementary materials

**Table S1. The names, isolate numbers, and corresponding GenBank accession numbers of the taxa used in Figure 1. The taxa produced in this study are indicated in red, and the type strains are indicated in bold with "T".**

| Species | Isolate No. | GenBank accession No. | |
|---|---|---|---|
| | | ITS | *tub2* |
| ***Allocryptovalsa cryptovalsoidea*** | **HVFIG02 T** | **HQ692573** | **HQ692524** |
| *Allocryptovalsa cryptovalsoidea* | HVFIG05 | HQ692574 | HQ692525 |
| ***Allocryptovalsa polyspora*** | **MFLUCC 17-0364 T** | **MF959500** | **MG334556** |
| *Allocryptovalsa rabenhorstii* | WA07CO | HQ692620 | HQ692522 |
| *Allocryptovalsa rabenhorstii* | CreI | KC774567 | / |
| ***Allodiatrype arengae*** | **MFLUCC 15-0713 T** | **MN308411** | **MN340297** |
| *Allodiatrype elaeidicola* | MFLUCC 15-0737a | MN308415 | MN340299 |
| *Allodiatrype elaeidis* | MFLUCC 15-0708b | MN308413 | / |
| *Allodiatrype elaeidis* | MFLUCC 15-0708a | MN308412 | MN340298 |
| *Allodiatrype thailandica* | MFLUCC 15-0711 | MN308414 | / |
| *Anthostoma decipiens* | JL567 | JN975370 | JN975407 |
| ***Anthostoma decipiens*** | **IPV-FW349 T** | **AM399021** | **AM920693** |
| *Cryptosphaeria avicenniae* | NFCCI-4248 | MH304406 | MH370273 |
| ***Cryptosphaeria bathurstensis*** | **AMH-9952 T** | **MN061366** | **MN431496** |
| *Cryptosphaeria eunomia* | CBS 216.87 | AJ302417 | / |
| *Cryptosphaeria eunomia* | CBS223.87 | AJ302421 | / |
| *Cryptosphaeria ligniota* | CBS 273.87 | AJ302418 | KT425168 |
| *Cryptosphaeria moravica* | CBS244.87 | HM164735 | HM164769 |
| *Cryptosphaeria pullmanensis* | HBPF24 | KT425202 | GQ294014 |
| *Cryptosphaeria pullmanensis* | ATCC 52655 | KT425235 | KT425170 |
| *Cryptosphaeria subcutanea* | CBS 240.87 | AJ302420 | KT425167 |
| *Cryptosphaeria subcutanea* | DSUB100A | KT425189 | KT425124 |
| *Cryptovalsa ampelina* | UCD779St | GQ293907 | GQ293980 |
| *Cryptovalsa ampelina* | A001 | GQ293901 | GQ293972 |
| *Cryptovalsa ampelina* | DRO101 | GQ293902 | GQ293982 |
| *Cryptovalsa ampelina* | IRAN 2281C | KJ767718 | KY352426 |
| *Diatrype bullata* | UCDDCh400 | DQ006946 | DQ007002 |
| ***Diatrype disciformis*** | **CBS 205.87 T** | **AJ302437** | **/** |
| *Diatrype disciformis* | MFLUCC15-0538 | KR605644 | KY352434 |
| *Diatrype exteroxantha* | HUEFS155114 | KM396617 | KT003700 |
| *Diatrype exteroxantha* | HUEFS155116 | KM396618 | KT022236 |
| *Diatrype macowaniana* | CBS 214.87 | AJ302431 | / |
| *Diatrype mangrovei* | MFLUCC 17-0412 | MH304407 | / |

| | | | |
|---|---|---|---|
| *Diatrype mangrovei* | MFLUCC 17-0391 | MH304408 | / |
| *Diatrype mangrovei* | MFLUCC 17-0394 | MH304409 | / |
| ***Diatrype palmicola*** | **MFLU 15-0040 T** | **KP744439** | **/** |
| *Diatrype stigma* | DCASH200 | GQ293947 | GQ294003 |
| *Diatrype undulate* | CBS 271.87 | AJ302436 | / |
| *Diatrypella favaceae* | Isolate 380 | KU320616 | / |
| *Diatrypella pulvinata* | H048 | FR715523 | FR715495 |
| *Eutypa astroidea* | CBS 292.87 | AJ302458 | DQ006966 |
| *Eutypa cremea* | STEU 8087 | KY111646 | KY111603 |
| *Eutypa cremea* | STEU 8082 | KY111656 | KY111598 |
| *Eutypa laevata* | CBS 291.87 | HM164737 | HM164771 |
| *Eutypa lata* | SAPN01 | HQ692616 | HQ692500 |
| *Eutypa lata va. aceri* | CBS 290.87 | HM164736 | HM164770 |
| *Eutypa lejoplaca* | 020202-5 | AY684221 | AY684196 |
| *Eutypa leptoplaca* | CBS 287.87 | AY684226 | AY684204 |
| *Eutypa leptoplaca* | CBS 288.88 | AY684227 | AY684205 |
| *Eutypa sparsa* | 3802-3b | AY684220 | AY684201 |
| *Eutypella caricae* | EL51C | AJ302460 | / |
| *Eutypella cerviculata* | CBS 221.87 | AJ302468 | / |
| ***Eutypella cerviculata*** | **M68 T** | **JF340269** | **/** |
| *Eutypella leprosa* | EL54C | AJ302463 | / |
| *Eutypella leprosa* | Isolate 60 | KU320622 | / |
| *Eutypella microtheca* | BCMX01 | KC405563 | KC405560 |
| *Eutypella microtheca* | ADEL200 | HQ692559 | HQ692527 |
| *Halodiatrype avicenniae* | MFLUCC 16-0532 | MH304411 | MH370275 |
| *Halodiatrype avicenniae* | MFLUCC 17-0396 | MH304413 | MH370277 |
| *Halodiatrype avicenniae* | MFLUCC 15-0948 | MH304414 | MH370278 |
| *Halodiatrype avicenniae* | MFLUCC 16-0533 | MH304412 | MH370276 |
| ***Halodiatrype salinicola*** | **MFLUCC15-1277 T** | **KX573915** | **KX573932** |
| *Kretzschmaria deusta* | CBS 826.72 | KU683767 | KU684190 |
| *Libertella blepharis* | LBAg | AY620998 | / |
| *Libertella* sp. | F6 | OL336844 | / |
| ***Mangifericola hongheensis*** | **HKAS 122665 T** | **OM030351** | **ON468664** |
| *Mangifericola hongheensis* | HKAS 122666 | OM030348 | ON468665 |
| ***Melanostictus longiostiolatus*** | **MFLU 19-2146 T** | **NR_175675** | **MW775595** |
| ***Melanostictus thailandicus*** | **MFLU 19-2123 T** | **NR_175671** | **MW775590** |
| *Monosporascus cannonballus* | CMM3646 | JX971617 | / |
| ***Monosporascus cannonballus*** | **ATCC 26931 T** | **FJ430598** | **/** |
| *Neoeutypella baoshanensis* | GMB:0052 | MW797106 | MW814878 |
| ***Neoeutypella baoshanensis*** | **HMAS 255436 T** | **NR_164038** | **MH822888** |

| | | | |
|---|---|---|---|
| *Paraeutypella citricola* | IRAN 2349C | KR605647 | KY352439 |
| *Paraeutypella citricola* | KUMCC 21-0461 | OL989101 | ON468663 |
| *Paraeutypella citricola* | HVVIT07 | HQ692579 | HQ692512 |
| *Paraeutypella citricola* | HVGRF01 | HQ692589 | HQ692521 |
| *Paraeutypella citricolca* | KUMCC 20-0023 | MW040050 | MW239663 |
| *Paraeutypella guizhouensis* | KUMCC 20-0017 | MW039348 | MW239661 |
| ***Paraeutypella guizhouensis*** | **KUMCC 20-0016 T** | **MW036142** | **MW239660** |
| *Paraeutypella quatemata* | CBS 196.30 | MH855110 | / |
| *Paraeutypella vitis* | UCD2291AR | HQ288224 | HQ288303 |
| *Paraeutypella vitis* | UCD2428TX | FJ790851 | GU294726 |
| *Pedumispora rhizophorae* | BCC44877 | KJ888854 | / |
| *Pedumispora rhizophorae* | BCC44878 | KJ888853 | / |
| *Peroneutypa alsophila* | CBS 250.87 | AJ302467 | / |
| *Peroneutypa comosa* | BAFC 393 | KF964568 | / |
| *Peroneutypa curvispora* | HUEFS 136877 | KM396641 | / |
| ***Peroneutypa diminutispora*** | **HUEFS 192196 T** | **KM396647** | **/** |
| *Peroneutypa kochiana* | EL53M | AJ302462 | / |
| ***Peroneutypa microasca*** | **BAFC 51550 T** | **KF964566** | **KF964572** |
| ***Peroneutypa rubiformis*** | **MFLUCC 17-2142 T** | **MG873477** | **/** |
| *Peroneutypa scoparia* | DFMAL100 | GQ293962 | GQ294029 |
| *Peroneutypa scoparia* | IRAN 2345C | KR605646 | KY352452 |
| *Quaternaria quaternata* | CBS 278.87 | AJ302469 | / |
| *Quaternaria quaternata* | IRAN 2348C | KR605645 | KY352464 |
| ***Xylaria berteroi*** | **YMJ 95101511 T** | **KC473562** | **KC473561** |

**Table S2. The names, isolate numbers, and corresponding GenBank accession numbers of the taxa used in figure 3. The taxa produced in this study are indicated in red, and the type strains are indicated in bold with "T".**

| Species name | Isolate No. | GenBank accession No. | | | |
|---|---|---|---|---|---|
| | | ITS | LSU | *rpb2* | *tub2* |
| *Annulohypoxylon annulatum* | CBS 140775 | KY610418 | KY610418 | KY624263 | KX376353 |
| *Annulohypoxylon truncatum* | CBS 140778 | KY610419 | KY610419 | KY624277 | KX376352 |
| *Biscogniauxia nummularia* | MUCL 51395 | KY610382 | KY610427 | KY624236 | KX271241 |
| *Daldinia concentrica* | CBS 113277 | AY616683 | KY610434 | KY624243 | KC977274 |
| ***Daldinia dennisii*** | **CBS 114741 T** | **JX658477** | **KY610435** | **KY624244** | **KC977262** |
| *Daldinia loculatoides* | CBS 113279 | AF176982 | KY610438 | KY624247 | KX271246 |
| *Daldinia steglichii* | MUCL 43512 | KY610399 | KY610479 | KY624250 | KX271269 |
| *Entonaema liquescens* | ATCC 46302 | KY610389 | KY610443 | KY624253 | KX271248 |
| *Htfpoxtflon eurasiaticum* | MUCL 57722 | MW367853 | / | MW373854 | MW373863 |
| ***Hypomontagnella barbarensis*** | **STMA 14081 T** | **MK131720** | **MK131718** | **MK135891** | **MK135893** |
| *Hypomontagnella monticulosa* | MUCL 54604 | KY610404 | KY610487 | KY624305 | KX271273 |
| *Hypomontagnella monticulosa* | CLL 205 | MK131719 | MK131717 | MK135890 | MK135892 |
| *Hypomontagnella monticulosa* | HKAS 122664 | OL989326 | OM001328 | ON392010 | ON468657 |
| *Hypomontagnella submonticulosa* | CBS 115280 | KC968923 | KY610457 | KY624226 | KC977267 |
| ***Hypoxylon addis*** | **MUCL 52797 T** | **KC968931** | / | / | **KC968931** |
| *Hypoxylon anthochroum* | YMJ 9 | JN660819 | / | / | AY951703 |
| ***Hypoxylon aurantium*** | **MFLU 16-1202 T** | **MN047114** | **NG_068298** | / | / |
| *Hypoxylon aurantium* | MFLU 18-0531 | MN047115 | MN017879 | MN077081 | / |
| ***Hypoxylon aveirense*** | **CMG 29 T** | **MN053021** | / | / | **MN066636** |
| ***Hypoxylon baihualingense*** | **FCATAS 477 T** | **MG490190** | / | / | **MH790276** |
| *Hypoxylon baruense* | UCH9545 | MN056428 | / | / | MK908142 |

| | | | | | |
|---|---|---|---|---|---|
| *Hypoxylon begae* | YMJ 215 | JN660820 | / | / | AY951704 |
| *Hypoxylon bellicolor* | UCH9543 | MN056425 | / | / | MK908139 |
| *Hypoxylon brevisporum* | YMJ 36 | JN660821 | / | / | AY951705 |
| *Hypoxylon carneum* | MUCL 54177 | KY610400 | KY610480 | KY624297 | KX271270 |
| *Hypoxylon cercidicola* | CBS 119009 | KC968908 | KY610444 | KY624254 | KC977263 |
| ***Hypoxylon chrysalidosporum*** | **FCATAS 2710 T** | **OL467294** | **OL615106** | **OL584222** | **OL584229** |
| *Hypoxylon crocop eplum* | CBS 119004 | KC968907 | KY610445 | KY624255 | KC977268 |
| *Hypoxylon crocopeplum* | ANM 1118 | JN673047 | / | / | / |
| ***Hypoxylon cyclobalanopsidis*** | **FCATAS 2714 T** | **OL467298** | **OL615108** | **OL584225** | **OL584232** |
| ***Hypoxylon damuense*** | **FCATAS4207 T** | **ON075427** | **ON075433** | **ON093251** | **ON093245** |
| ***Hypoxylon delonicis*** | **MFLU 16-1031 T** | **NR_171100** | **NG_074451** | **/** | **MT212215** |
| *Hypoxylon dieckmannii* | YMJ 89041203 | JN979413 | / | / | AY951713 |
| *Hypoxylon duranii* | YMJ 85 | JN979414 | / | / | AY951714 |
| *Hypoxylon erythrostroma* | YMJ 90080602 | JN979416 | / | / | AY951716 |
| *Hypoxylon eurasiaticum* | MUCL 57720 | MW367851 | / | MW373852 | MW373861 |
| *Hypoxylon eurasiaticum* | MUCL 57721 | MW367852 | / | MW373853 | MW373862 |
| *Hypoxylon eurasiaticum* | MUCL 57723 | MW367854 | / | MW373855 | MW373864 |
| *Hypoxylon eurasiaticum* | DSM 112037 | MW367855 | / | MW373856 | MW373865 |
| *Hypoxylon fendleri* | MUCL 54792 | KF234421 | KY610481 | KY624298 | KF300547 |
| *Hypoxylon ferrugineum* | CBS 141259 | KX090079 | / | / | KX090080 |
| *Hypoxylon fragiforme* | MUCL 51264 | KC477229 | KM186295 | KM186296 | KX271282 |
| *Hypoxylon fraxinophilum* | MUCL 54176 | KC968938 | / | / | KC977301 |
| ***Hypoxylon fulvosulphureum*** | **MFLUCC13-0589 T** | **KP401576** | **/** | **/** | **KP401584** |
| *Hypoxylon fuscum* | DSM 112039 | MW367856 | MW367847 | MW373857 | MW373866 |
| *Hypoxylon fuscum* | CBS 113049 | KY610401 | KY610482 | KY624299 | KX271271 |
| ***Hypoxylon griseobrunneum*** | **CBS 331.73 T** | **KY610402** | **KY610483** | **KY624300** | **KC977303** |

| | | | | | |
|---|---|---|---|---|---|
| ***Hypoxylon guilanense*** | **MUCL 57726 T** | **MT214997** | **MT214992** | **MT212235** | **MT212239** |
| *Hypoxylon haematostroma* | MUCL 53301 | KC968911 | KY610484 | KY624301 | KC977291 |
| ***Hypoxylon hepaticolor*** | **ILLS:00121426 T** | **MT799854** | **MT799853** | / | / |
| *Hypoxylon hinnuleum* | DSM:107926 | MK287532 | MK287544 | MK287557 | MK287570 |
| ***Hypoxylon hinnuleum*** | **MUCL:3621 T** | **MK287537** | **MK287549** | **MK287562** | **MK287575** |
| *Hypoxylon hongheensis* | KUMCC 21-0452 | OM001333 | OM001334 | ON39008 | ON468655 |
| ***Hypoxylon hongheensis*** | **HKAS 122663 T** | **OM001336** | **OM001339** | **ON392009** | **ON468656** |
| *Hypoxylon howeanum* | MUCL 47599 | AM749928 | KY610448 | KY624258 | KC977277 |
| *Hypoxylon hypomiltum* | MUCL 51845 | KY610403 | KY610449 | KY624302 | KX271249 |
| ***Hypoxylon invadens*** | **MUCL 51475 T** | **MT809133** | **MT809132** | **MT813037** | **MT813038** |
| *Hypoxylon investiens* | CBS 118183 | KC968925 | KY610450 | KY624259 | KC977270 |
| ***Hypoxylon isabellinum*** | **STMA 10247 T** | **KC968935** | / | / | **KC977295** |
| *Hypoxylon jecorinum* | YMJ 39 | JN979429 | / | / | AY951731 |
| ***Hypoxylon jianfengense*** | **FACATAS845 T** | **MW984546** | **MZ029707** | **MZ047260** | **MZ047264** |
| ***Hypoxylon larissae*** | **FACATAS844 T** | **MW984548** | **MZ029706** | **MZ047258** | **MZ047262** |
| ***Hypoxylon lateripigmentum*** | **MUCL 53304 T** | **KC968933** | **KY610486** | **KY624304** | **KC977290** |
| *Hypoxylon lenormandii* | CBS 119003 | KC968943 | KY610452 | KY624261 | KC977273 |
| *Hypoxylon lienhwacheense* | MFLUCC 14-1231 | KU604558 | MK287550 | MK287563 | KU159522 |
| ***Hypoxylon lignicola*** | **MFLUCC 16-0926 T** | **MK828609** | **MK835808** | / | / |
| *Hypoxylon liviae* | CBS 115282 | NR155154 | / | / | KC977265 |
| *Hypoxylon lividicolor* | YMJ 70 | JN979432 | / | / | AY951734 |
| *Hypoxylon lividipigmentum* | YMJ 233 | JN979433 | / | / | AY951735 |
| *Hypoxylon macrosporum* | YMJ 47 | JN979434 | / | / | AY951736 |
| ***Hypoxylon mangrovei*** | **MFLU 18-0575 T** | **MN047117** | **MN017881** | / | **MN077054** |
| ***Hypoxylon medogense*** | **FCATAS4061 T** | **ON075425** | **ON075431** | **ON093249** | **ON093243** |
| *Hypoxylon musceum* | MUCL 53765 | KC968926 | KY610488 | KY624306 | KC977280 |

| | | | | | |
|---|---|---|---|---|---|
| *Hypoxylon notatum* | YMJ 250 | JQ009305 | / | / | AY951739 |
| *Hypoxylon ochraceum* | MUCL 54625 | KC968937 | / | KY624271 | KC977300 |
| ***Hypoxylon olivaceopigmentum*** | **DSM 107924 T** | **MK287530** | **MK287542** | **MK287555** | **MK287568** |
| ***Hypoxylon papillatum*** | **ATCC 58729 T** | **KC968919** | **KY610454** | **KY624223** | **KC977258** |
| *Hypoxylon perforatum* | CBS 115281 | KY610391 | KY610455 | KY624224 | KX271250 |
| ***Hypoxylon petriniae*** | **CBS 114746 T** | **KY610405** | **KY610491** | **KY624279** | **KX271274** |
| *Hypoxylon pilgerianum* | STMA 13455 | KY610412 | KY610412 | KY624308 | KY624315 |
| *Hypoxylon porphyreum* | CBS 119022 | KC968921 | KY610456 | KY624225 | KC977264 |
| *Hypoxylon pseudofendleri* | MFLUCC 11-0639 | KU940156 | KU863144 | / | / |
| *Hypoxylon pseudofuscum* | DSM 112035 | MW367858 | MW367849 | MW373859 | MW373868 |
| *Hypoxylon pseudofuscum* | DSM 112036 | MW367859 | MW367850 | MW373860 | MW373869 |
| ***Hypoxylon pseudofuscum*** | **DSM 112038 T** | **MW367857** | **MW367848** | **MW373858** | **MW373867** |
| ***Hypoxylon pulicicidum*** | **CBS 122622 T** | **JX183075** | **KY610492** | **KY624280** | **JX183072** |
| *Hypoxylon rickii* | MUCL 53309 | KC968932 | KY610416 | KY624281 | KC977288 |
| *Hypoxylon rubiginosum* | MUCL 52887 | KC477232 | KY610469 | KY624266 | KY624311 |
| *Hypoxylon rutilum* | YMJ 181 | / | / | / | AY951752 |
| *Hypoxylon samuelsii* | MUCL 51843 | KC968916 | KY610466 | KY624269 | KC977286 |
| *Hypoxylon shearii* | YMJ 29 | EF026142 | / | / | AY951753 |
| ***Hypoxylon spegazzinianum*** | **STMA 14082 T** | **KU604573** | **/** | **/** | **KU604582** |
| ***Hypoxylon sporistriatatunicum*** | **UCH9542 T** | **MN056426** | **/** | **/** | **MK908140** |
| *Hypoxylon subgilvum* | YMJ 88113007 | JQ009315 | / | / | AY951755 |
| ***Hypoxylon sublenormandii*** | **JF 13026 T** | **KM610291** | **/** | **/** | **KM610303** |
| *Hypoxylon teeravasati* | PUFD4 | KY863509 | MF385274 | MG986895 | MG986894 |
| *Hypoxylon texense* | DSM:107928 | MK287527 | MK287538 | MK287551 | MK287564 |
| ***Hypoxylon texense*** | **DSM 107933 T** | **MK287536** | **MK287548** | **MK287561** | **MK287574** |
| *Hypoxylon ticinense* | CBS 115271 | JQ009317 | KY610471 | KY624272 | AY951757 |

| | | | | | |
|---|---|---|---|---|---|
| *Hypoxylon trugodes* | MUCL 54794 | KF234422 | KY610493 | KY624282 | KF300548 |
| *Hypoxylon ulmophilum* | YMJ 350 | JQ009320 | / | / | AY951760 |
| *Hypoxylon vogesiacum* | CBS 115273 | KC968920 | KY610417 | KY624283 | KX271275 |
| ***Hypoxylon wujiangense*** | **GMBC0213 T** | **MT568854** | **MT568853** | **MT585802** | **MT572481** |
| ***Hypoxylon wuzhishanense*** | **FCATAS2708 T** | **OL467292** | **OL615104** | **OL584220** | **OL584227** |
| ***Hypoxylon zangii*** | **FCATAS4029 T** | **ON075423** | **ON075429** | **ON093247** | **ON093241** |
| *Jackrogersella cohaerens* | CBS 119126 | KY610396 | KY610497 | KY624270 | KY624314 |
| *Jackrogersella multiformis* | CBS 119016 | KC477234 | KY610473 | KY624290 | KX271262 |
| *Pyrenopolyporus hunteri* | MUCL 52673 | KY610421 | KY610472 | KY624309 | KU159530 |
| ***Pyrenopolyporus laminosus*** | **MUCL 53305 T** | **KC968934** | **KY610485** | **KY624303** | **KC977292** |
| *Rhopalostroma angolense* | CBS 126414 | KY610420 | KY610459 | KY624228 | KX271277 |
| ***Rostrohypoxylon terebratum*** | **CBS 119137 T** | **DQ631943** | **DQ840069** | **DQ631954** | **DQ840097** |
| ***Ruwenzoria pseudoannulata*** | **MUCL 51394 T** | **KY610406** | **KY610494** | **KY624286** | **KX271278** |
| ***Thamnomyces dendroidea*** | **CBS 123578 T** | **FN428831** | **KY610467** | **KY624232** | **KY624313** |
| *Xylaria hypoxylon* | CBS 122620 | KY610407 | KY610495 | KY624231 | KX271279 |

**Table S3. The names, isolate numbers, and corresponding GenBank accession numbers of the taxa used in Figure 5. The taxa produced in this study are indicated in red, and the type strains are indicated in bold with "T".**

| **Species name** | **Isolate No.** | **GenBank accession No.** | | | |
|---|---|---|---|---|---|
| | | **ITS** | ***tef1-α*** | ***tub2*** | **CAL** |
| *Diaporthe acuta* | PSCG 046 | MK626958 | / | MK691224 | MK691125 |
| ***Diaporthe acutispora*** | **LC6161 T** | **KX986764** | **KX999155** | **KX999195** | **KX999274** |
| ***Diaporthe arecae*** | **CBS 161.64 T** | **KC343032** | **KC343758** | **KC344000** | **KC343274** |
| ***Diaporthe arengae*** | **CBS 114979 T** | **NR_111843** | **KC343760** | **KC344002** | **KC343276** |
| ***Diaporthe aseana*** | **MFLUCC 12-0299a T** | **NR_154920** | **KT459448** | **KT459432** | **KT459464** |
| ***Diaporthe australiana*** | **BRIP 66145 T** | **MN708222** | **MN696522** | **MN696530** | **/** |
| *Diaporthe biconispora* | ZJUD62 | KJ490597 | KJ490476 | KJ490418 | MT898460 |
| ***Diaporthe ceratozamiae*** | **CBS 131306 T** | **JQ044420** | **/** | **/** | **/** |
| *Diaporthe cercidis* | TJX65 | MW341306 | MW362968 | MW491981 | MW491970 |
| ***Diaporthe endocitricola*** | **ZHKUCC20-0013 T** | **MT355683** | **MT409337** | **MT409291** | **MT409313** |
| *Diaporthe eucalyptorum* | MFLUCC 12-0306 | KT459419 | KT459453 | KT459437 | / |
| *Diaporthe eugeniae* | ASHM302 | MK110368 | MK117249 | MK122781 | / |
| *Diaporthe eugeniae* | ASHM298 | MK110346 | MK117246 | MK122779 | / |
| ***Diaporthe eugeniae*** | **CBS 444.82 T** | **KC343098** | **KC343824** | **KC344066** | **KC343340** |
| *Diaporthe fraxini-angustifoliae* | MFLUCC 15-0748 | KT459428 | KT459446 | KT459430 | KT459462 |
| *Diaporthe fujianensis* | JZB320152 | MW010215 | MW205233 | MW056011 | / |
| *Diaporthe fusiformis* | JZB320157 | MW010219 | / | MW056015 | / |
| *Diaporthe ganzhouensis* | CFCC 53087 | MK432665 | MK578139 | MK578065 | MK442985 |

| | | | | | |
|---|---|---|---|---|---|
| ***Diaporthe guangxiensis*** | **JZB320094 T** | **MK335772** | **MK523566** | **MK500168** | **MK736727** |
| *Diaporthe heveae* | LGMF1631 | MG976433 | MK007529 | MK007530 | / |
| *Diaporthe hongheensis* | KUMCC 21-0458 | OM001330 | ON468650 | ON468659 | ON715009 |
| ***Diaporthe hongheensis*** | **KUMCC 21-0457 T** | **OM001331** | **ON468649** | **ON468658** | **ON715010** |
| *Diaporthe hongkongensis* | ZJ10B1 | KY433562 | KY433571 | / | / |
| *Diaporthe limonicola* | ZHKUCC20-0005 | MT355675 | MT409329 | MT409283 | MT409306 |
| *Diaporthe limonicola* | ZHKUCC20-0006 | MT355676 | MT409330 | MT409284 | MT409307 |
| ***Diaporthe limonicola*** | **CPC 28200 T** | **MF418422** | **MF418501** | **MF418582** | **MF418256** |
| ***Diaporthe litchicola*** | **BRIP 54900 T** | **JX862533** | **JX862539** | **KF170925** | **/** |
| ***Diaporthe lithocarpus*** | **CGMCC:3.15175 T** | **KC153104** | **KC153095** | **KF576311** | **KF576236** |
| ***Diaporthe melitensis*** | **CPC 27873 T** | **MF418424** | **MF418503** | **MF418584** | **MF418258** |
| ***Diaporthe millettia*** | **GUCC 9167 T** | **MK303387** | **MK480609** | **MK460488** | **MK502086** |
| *Diaporthe multigutullata* | CFCC 53095 | MK432645 | MK578121 | MK578048 | MK442967 |
| *Diaporthe multigutullata* | CFCC 53096 | MK432646 | MK578122 | MK578049 | MK442968 |
| ***Diaporthe multigutullata*** | **ZJUD98 T** | **NR_158389** | **KJ490512** | **KJ490454** | **/** |
| ***Diaporthe musigena*** | **CBS 129519 T** | **KC343143** | **KC343869** | **KC344111** | **KC343385** |
| *Diaporthe osmanthusis* | GUCC9165 | MK398675 | MK480610 | MK502091 | MK502087 |
| ***Diaporthe pandanicola*** | **MFLU 17-0607 T** | **NR_172400** | **/** | **MG646930** | **/** |
| *Diaporthe pascoei* | BPPCA147 | MK111091 | MK117255 | MK122790 | / |
| *Diaporthe pascoei* | PBMR343 | MK111092 | MK117274 | MK122808 | / |
| ***Diaporthe pascoei*** | **BRIP 54847 T** | **JX862532** | **JX862538** | **KF170924** | **/** |
| ***Diaporthe perjuncta*** | **CBS 109745 T** | **NR_147527** | **KC343898** | **KC344140** | **KC343414** |
| ***Diaporthe perseae*** | **CBS 151.73 T** | **KC343173** | **KC343899** | **KC344141** | **KC343415** |

| | | | | | |
|---|---|---|---|---|---|
| ***Diaporthe pescicola*** | **MFLUCC 16-0105 T** | **KU557555** | **KU557623** | **KU557579** | **KU557603** |
| *Diaporthe phoenicicola* | CBS 161.64 | MH858400 | GQ250349 | X275440 | JX197432 |
| ***Diaporthe phragmitis*** | **CBS 138897 T** | **KP004445** | **/** | **KP004507** | **/** |
| *Diaporthe podocarpi-macrophylli* | LC6197 | KX986777 | KX999170 | KX999210 | KX999279 |
| *Diaporthe podocarpi-macrophylli* | CQJY3-1 | MT877048 | MT917080 | MT874966 | MT917067 |
| ***Diaporthe pseudomangiferae*** | **CBS 101339 T** | **NR_111858** | **KC343907** | **KC344149** | **KC343423** |
| ***Diaporthe pseudophoenicicola*** | **CBS 462.69 T** | **KC343184** | **KC343910** | **KC344152** | **KC343426** |
| ***Diaporthe pterocarpi*** | **MFLUCC 10-0571 T** | **JQ619899** | **JX275416** | **JX275460** | **JX275416** |
| ***Diaporthe pterocarpicola*** | **MFLUCC 10-0580a T** | **NR_111713** | **JX275403** | **JX275441** | **JX197433** |
| *Diaporthe salsuginosa* | NFCCI 4385 | MN061372 | MN184789 | MN431500 | / |
| *Diaporthe schimae* | CFCC 53103 | MK432640 | MK578116 | MK578043 | MK442962 |
| *Diaporthe schimae* | CFCC 53104 | MK432641 | MK578117 | MK578044 | MK442963 |
| ***Diaporthe sennae*** | **CFCC 51636 T** | **NR_152499** | **KY228885** | **KY228891** | **KY228875** |
| *Diaporthe sennae* | ZHKUCC20-0011 | / | MT409335 | MT409289 | MT409311 |
| *Diaporthe* sp. | MFLUCC 19-0102 | MW192408 | MW173088 | / | MW294202 |
| *Diaporthe taiwanensis* | NTUCC 18-105.2 | MT241259 | MT251201 | MT251204 | MT251198 |
| *Diaporthe taiwanensis* | NTUCC 18-105.1 | MT241257 | MT251199 | MT251202 | MT251196 |
| ***Diaporthe taoicola*** | **MFLUCC 16-0117 T** | **NR_154923** | **KU557635** | **KU557591** | **/** |
| ***Diaporthe tectonigena*** | **MFLUCC 12-0767 T** | **NR_147589** | **KU749371** | **KU743976** | **KU749358** |
| ***Diaporthe undulata*** | **LC8111 T** | **KY491546** | **KY491556** | **KY491566** | **/** |
| ***Diaporthe vawdreyi*** | **BRIP 57887a T** | **KR936126** | **KR936129** | **KR936128** | **/** |
| *Diaporthe viniferae* | JZB320072 | MK341551 | MK500108 | MK500113 | MK500120 |
| ***Diaporthe viniferae*** | **JZB320071 T** | **MK341550** | **MK500107** | **MK500112** | **MK500119** |

| | | | | | |
|---|---|---|---|---|---|
| ***Diaporthe xishuangbanica*** | **LC6707 T** | **KX986783** | **KX999175** | **KX999216** | **/** |
| *Diaporthe xunwuensis* | CFCC 53085 | MK432663 | MK578137 | MK578063 | MK442983 |
| *Diaporthe xunwuensis* | CFCC 53086 | MK432664 | MK578138 | MK578064 | MK442984 |
| *Diaporthella corylina* | CBS 121124 | KC343004 | KC343730 | KC343972 | KC343246 |

**Table S4. The names, isolate numbers, and corresponding GenBank accession numbers of the taxa used in Figure 7. The taxa produced in this study are indicated in red, and the type strains are indicated in bold with "T".**

| **Speciesname** | **Isolate No.** | **GenBank Accession No** | | | |
|---|---|---|---|---|---|
| | | **ITS** | **LSU** | **SSU** | ***rpb1*** |
| *Cyphellophora aestiva* | CBS 227.86 | JQ766425 | JQ766474 | / | JQ766380 |
| ***Cyphellophora aestiva*** | **CBS 228.86 T** | **MH861947** | **MH873637** | **NG_062863** | **JQ766381** |
| *Cyphellophora ambigua* | CMRP2859 | MT075638 | / | / | / |
| *Cyphellophora artocarpi* | CHCJHBJBLM | KP010367 | KP122930 | / | KP122920 |
| ***Cyphellophora attinorum*** | **CBS 131958 T** | **KF928463** | **KF928527** | **/** | **/** |
| ***Cyphellophora capiguarae*** | **CBS 132767 T** | **KF928464** | **KF928528** | **/** | **/** |
| ***Cyphellophora clematidis*** | **CBS 144983 T** | **MK442577** | **MK442519** | **/** | **/** |
| ***Cyphellophora eucalypti*** | **CBS 124764 T** | **KC455238** | **KC455254** | **KC455297** | **/** |
| *Cyphellophora europaea* | CBS 101466 | KC455246 | KC455259 | KC455303 | / |
| *Cyphellophora filicis* | DP002A | MK404056 | MK404052 | MK404054 | / |
| ***Cyphellophora filicis*** | **DP002B T** | **MK404057** | **MK404053** | **MK404055** | **/** |
| *Cyphellophora fusarioides* | CBS 130291 | JQ766439 | JQ766486 | / | JQ766391 |

| | | | | | |
|---|---|---|---|---|---|
| ***Cyphellophora fusarioides*** | **MUCL 44033 T** | **KC455239** | **KC455252** | **KC455298** | **/** |
| ***Cyphellophora gamsii*** | **CPC 25867 T** | **KX228255** | **NG_067308** | **/** | **/** |
| ***Cyphellophora goniomatis*** | **CPC 37032 T** | **NR_166332** | **NG_068669** | **/** | **/** |
| *Cyphellophora guyanensis* | CBS 124764 | KF928477 | KF928541 | / | JQ766387 |
| *Cyphellophora guyanensis* | CBS 125756 | JQ766433 | JQ766482 | / | JQ766388 |
| *Cyphellophora guyanensis* | CBS 126014 | JQ766434 | JQ766483 | / | JQ766389 |
| *Cyphellophora guyanensis* | CBS 126020 | KF928475 | KF928539 | / | JQ766390 |
| *Cyphellophora hongheensis* | KUMCC 21-0456 | OM001332 | OM001329 | OM001337 | ON468647 |
| ***Cyphellophora hongheensis*** | **KUMCC 21-0455 T** | **OM001338** | **OM001335** | **OM001340** | **ON468646** |
| ***Cyphellophora jingdongensis*** | **IFRDCC 2659 T** | **MF285234** | **MF285236** | **MF285235** | **/** |
| ***Cyphellophora laciniata*** | **CBS 190.61 T** | **MH858019** | **FJ358239** | **FJ358307** | **FJ358370** |
| ***Cyphellophora livistonae*** | **CPC 19433 T** | NR_111824 | NG_042752 | **/** | **/** |
| Cyphellophora ludoviensis | CMRP1317 | KX434722 | KX583708 | / | / |
| *Cyphellophora musae* | GLGZXJ9B | / | KP122931 | / | KP122923 |
| ***Cyphellophora musae*** | **GLZJXJ41 T** | **/** | **KP122932** | **/** | **KP122922** |
| ***Cyphellophora olivacea*** | **CBS 123.74 T** | **KC455248** | **NG_067280** | **NG_062865** | **/** |
| *Cyphellophora oxyspora* | CBS 416.89 | MH862181 | MH873869 | KF155208 | / |
| ***Cyphellophora oxyspora*** | **CBS 698.73 T** | **MH860790** | **NG_067405** | **NG_062866** | **/** |
| *Cyphellophora pauciseptata* | CBS 284.85 | MH861880 | MH873568 | / | JQ766421 |
| ***Cyphellophora phyllostachydis*** | **HLHNZWYZZ08 T** | **KP010371** | **KP122933** | **/** | **KP122924** |
| *Cyphellophora pluriseptata* | CBS 109633 | JQ766430 | JQ766479 | / | / |
| ***Cyphellophora pluriseptata*** | **CBS 286.85 T** | **NR_111431** | **NG_067429** | **NG_062861** | **JQ766384** |
| ***Cyphellophora reptans*** | **CBS 113.85 T** | **NR_121346** | **NG_067426** | **NG_062867** | **/** |

| | | | | | |
|---|---|---|---|---|---|
| ***Cyphellophora sambuci*** | **CPC 39957 T** | **OK664711** | **OK663750** | **/** | **/** |
| *Cyphellophora sessilis* | CBS 238.93 | KF928459 | KC455264 | KC455309 | KC455289 |
| ***Cyphellophora sessilis*** | **CBS 243.85 T** | **MH861875** | **MH873561** | **KC455308** | **KC455288** |
| ***Cyphellophora suttonii*** | **CBS 449.91 T** | **KC455243** | **KC455256** | **KC455300** | **JQ922030** |
| ***Cyphellophora vermispora*** | **CBS 228.86 T** | **KC455244** | **KC455257** | **KC455301** | **JQ766381** |
| ***Cyphellophora vietnamensis*** | **CBS 146924 T** | **LR814107** | **LR814108** | **/** | **/** |
| *Exophiala equina* | CBS 127579 | MH864585 | MH876027 | / | / |
| ***Exophiala salmonis*** | **CBS 157.67 T** | **MH858932** | **MH870616** | **JN856020** | **/** |

**Table S5. The names, isolate numbers, and corresponding GenBank accession numbers of the taxa used in Figure 9. The taxa produced in this study are indicated in red, and the type strains are indicated in bold with "T".**

| **Species** | **Isolate No.** | **GenBank Accession No** | | |
|---|---|---|---|---|
| | | **ITS** | ***tef-a*** | ***tub2*** |
| *Botryosphaeria stevensii* | CMW 7060 | AY236955 | AY236904 | AY236933 |
| ***Botryosphaeria obtusa*** | **CBS 112555 T** | **AY259094** | **AY573220** | **/** |
| ***Lasiodiplodia acaciae*** | **CBS:136434 T** | **MT587421** | **MT592133** | **MT592613** |
| ***Lasiodiplodia americana*** | **CFCC50065 T** | **KP217059** | **KP217067** | **KP217075** |
| *Lasiodiplodia aquilariae* | CGMCC 3.18471 | KY783442 | KY848600 | / |
| ***Lasiodiplodia avicenniae*** | **CMW41467 T** | **KP860835** | **KP860680** | **KP860758** |
| *Lasiodiplodia avicenniae* | LAS199 | KU587957 | KU587868 | KU587868 |
| ***Lasiodiplodia avicenniarum*** | **MFLUCC17-2591 T** | **MK347777** | **MK340867** | **/** |
| *Lasiodiplodia brasiliensis* | CBS:120395 | MT587423 | MT592135 | MT592615 |
| *Lasiodiplodia brasiliensis* | CBS:115447 | MT587422 | MT592134 | MT592614 |

| | | | | |
|---|---|---|---|---|
| *Lasiodiplodia bruguierae* | CMW42480 | KP860832 | KP860677 | KP860755 |
| ***Lasiodiplodia bruguierae*** | **CMW 41470 T** | **KP860833** | **KP860678** | **KP860756** |
| *Lasiodiplodia caatinguensis* | IBL381 | KT154757 | KT154751 | KT154764 |
| ***Lasiodiplodia caatinguensis*** | **CMM 1325 T** | **KT154760** | **KT008006** | **KT154767** |
| *Lasiodiplodia chinensis* | CGMCC 3.18044 | KX499875 | KX499913 | KX499988 |
| *Lasiodiplodia chinensis* | CGMCC3.18066 | KX499899 | KX499937 | KX500012 |
| *Lasiodiplodia cinnamomi* | CFCC 51998 | MG866029 | MH236800 | MH236798 |
| *Lasiodiplodia cinnamomi* | CFCC 51997 | MG866028 | MH236799 | MH236797 |
| *Lasiodiplodia citricola* | IRAN1521C | GU945353 | GU945339 | KU887504 |
| ***Lasiodiplodia citricola*** | **IRAN1522C T** | **GU945354** | **GU945340** | **KU887505** |
| *Lasiodiplodia clavispora* | CGMCC 3.19595 | MK802165 | / | MK816338 |
| ***Lasiodiplodia clavispora*** | **CGMCC 3.19594 T** | **MK802166** | **/** | **MK816339** |
| *Lasiodiplodia crassispora* | WAC12533 | DQ103550 | DQ103557 | KU887506 |
| *Lasiodiplodia crassispora* | CMM 4583 | MG954353 | MG979519 | MG979551 |
| ***Lasiodiplodia crassispora*** | **CMW 13488 T** | **DQ103552** | **DQ103559** | **KU887507** |
| *Lasiodiplodia curvata* | CGMCC 3.18456 | KY783437 | KY848596 | KY848529 |
| *Lasiodiplodia curvata* | CGMCC 3.18476 | KY783443 | KY848601 | KY848532 |
| *Lasiodiplodia egyptiacae* | CBS:167.28 | MT587425 | MT592136 | MT592618 |
| ***Lasiodiplodia endophytica*** | **MFLUCC 18-1121 T** | **MK501838** | **MK584572** | **MK550606** |
| *Lasiodiplodia euphorbicola* | CMW33353 | KU887152 | KU887028 | KU887456 |
| *Lasiodiplodia euphorbicola* | CMW36077 | KU887157 | KU887035 | KU887473 |
| *Lasiodiplodia euphorbicola* | CMM 3609 | KU887149 | KU887026 | KU887455 |
| *Lasiodiplodia euphorbicola* | CMW 33350 | KU887187 | KU887063 | KU887494 |
| *Lasiodiplodia exigua* | BL184 | KJ638318 | KJ638337 | / |
| ***Lasiodiplodia exigua*** | **CBS 137785 T** | **NR_147353** | **KJ638336** | **KU887509** |
| ***Lasiodiplodia fujianensis*** | **CGMCC 3.19593 T** | **MK802164** | **MK887178** | **MK816337** |

| | | | | |
|---|---|---|---|---|
| *Lasiodiplodia gilanensis* | IRAN 1523C | GU945352 | GU945341 | KU887510 |
| ***Lasiodiplodia gilanensis*** | **IRAN 1501C T** | **GU945351** | **GU945342** | **KU887511** |
| *Lasiodiplodia gonubiensis* | CMW43763 | KU587955 | KU587944 | KU587865 |
| *Lasiodiplodia gonubiensis* | CMW43762 | KU587954 | KU587943 | KU587864 |
| *Lasiodiplodia gonubiensis* | CMW 14078 | AY639594 | DQ103567 | EU673126 |
| ***Lasiodiplodia gonubiensis*** | **CMW 14077 T** | **AY639595** | **DQ103566** | **DQ458860** |
| *Lasiodiplodia gravistriata* | CMM 4565 | KT250947 | KT266812 | / |
| *Lasiodiplodia gravistriata* | CMM 4564 | KT250949 | KT250950 | / |
| *Lasiodiplodia henanica* | CGMCC 3.19176 | MH729351 | MH729357 | MH729360 |
| *Lasiodiplodia hormozganensis* | CBS:133510 | MT587426 | MT592137 | MT592620 |
| *Lasiodiplodia hormozganensis* | CBS:177.89 | KX464134 | KX464627 | KX464898 |
| *Lasiodiplodia hormozganensis* | IRAN 1498C | GU945356 | GU945344 | KU887514 |
| *Lasiodiplodia hyalina* | BJFU DZP160121-9 | / | KY751302 | KY751299 |
| ***Lasiodiplodia hyalina*** | **CGMCC3.17975 T** | **KX499879** | **KX499917** | **KX499992** |
| ***Lasiodiplodia indica*** | **IBP 01 T** | **KM376151** | **/** | **/** |
| *Lasiodiplodia iraniensis* | IRAN 1502C | GU945347 | GU945335 | KU887517 |
| *Lasiodiplodia iraniensis* | IRAN 1520C | GU945348 | GU945336 | KU887516 |
| ***Lasiodiplodia irregularis*** | **GuoLD01673 T** | **KY783472** | **KY848610** | **KY848553** |
| *Lasiodiplodia jatrophicola* | CMW 36237 | KU887121 | KU886998 | KU887499 |
| *Lasiodiplodia jatrophicola* | CMW 36239 | KU887123 | KU887000 | KU887501 |
| *Lasiodiplodia jatrophicola* | CBS:111005 | MT587430 | MT592142 | MT592624 |
| *Lasiodiplodia jatrophicola* | CBS:111008 | MT587431 | MT592143 | MT592625 |
| ***Lasiodiplodia krabiensis*** | **MFLU 17-2617 T** | **MN047093** | **MN077070** | **/** |
| *Lasiodiplodia laeliocattleyae* | BOT 29 | JN814401 | JN814428 | / |
| *Lasiodiplodia laeliocattleyae* | CMW35895 | KU887095 | KU886973 | KU887467 |
| ***Lasiodiplodia laeliocattleyae*** | **CBS 130992 T** | **JN814397** | **JN814424** | **KU887508** |

| | | | | |
|---|---|---|---|---|
| *Lasiodiplodia laosensis* | CGMCC 3.18473 | KY783450 | KY848603 | KY848536 |
| ***Lasiodiplodia laosensis*** | **CGMCC 3.18464 T** | **KY783471** | **KY848609** | **KY848552** |
| *Lasiodiplodia lignicola* | CGMCC 3.18449 | KY783466 | KY848619 | / |
| *Lasiodiplodia lignicola* | CBS 134112 | / | KU887003 | KT852958 |
| *Lasiodiplodia lignicola* | MFLUCC 11-0656 | JX646798 | JX646863 | JX646846 |
| ***Lasiodiplodia macroconidica*** | **CGMCC 3.18479 T** | **KY783438** | **KY848597** | **KY848530** |
| ***Lasiodiplodia macrospora*** | **CMM 3833 T** | **KF234557** | **KF226718** | **KF254941** |
| *Lasiodiplodia magnoliae* | MFLUCC 18-0948 | MK499387 | MK568537 | MK521587 |
| *Lasiodiplodia mahajangana* | CMW 27818 | FJ900596 | FJ900642 | FJ900631 |
| ***Lasiodiplodia mahajangana*** | **CMW 27801 T** | **FJ900595** | **FJ900641** | **FJ900630** |
| *Lasiodiplodia margaritacea* | CBS 138291 | KP872322 | KP872351 | KP872381 |
| *Lasiodiplodia margaritacea* | CBS 138289 | KP872320 | KP872349 | KP872379 |
| ***Lasiodiplodia margaritacea*** | **CBS 122519 T** | **EU144050** | **EU144065** | **KU887520** |
| *Lasiodiplodia mediterranea* | CBS 137784 | KJ638311 | KJ638330 | KU887522 |
| ***Lasiodiplodia mediterranea*** | **CBS 137783 T** | **KJ638312** | **KJ638331** | **KU887521** |
| ***Lasiodiplodia microcondia*** | **CGMCC 3.18485 T** | **KY783441** | **KY848614** | **/** |
| *Lasiodiplodia missouriana* | UCD 2193MO | HQ288225 | HQ288267 | HQ288304 |
| ***Lasiodiplodia missouriana*** | **UCD 2199MO T** | **HQ288226** | **HQ288268** | **HQ288305** |
| ***Lasiodiplodia mitidjana*** | **ALG111 T** | **MN104115** | **/** | **/** |
| *Lasiodiplodia nanpingensis* | CGMCC 3.19596 | MK802167 | / | MK816340 |
| *Lasiodiplodia nanpingensis* | CGMCC 3.19597 | MK802168 | / | MK816341 |
| ***Lasiodiplodia pandanicola*** | **MFLUCC 16-0265 T** | **MH275068** | **MH412774** | **/** |
| *Lasiodiplodia paraphysoides* | CGMCC 3.19174 | MH729349 | MH729355 | MH729358 |
| *Lasiodiplodia paraphysoides* | CGMCC 3.19175 | MH729350 | MH729356 | MH729359 |
| *Lasiodiplodia parva* | CBS 494.78 | EF622084 | EF622064 | EU673114 |
| ***Lasiodiplodia parva*** | **CBS 456.78 T** | **EF622083** | **EF622063** | **KU887523** |

| | | | | |
|---|---|---|---|---|
| *Lasiodiplodia plurivora* | STE-U 4583 | AY343482 | EF445396 | KU887525 |
| *Lasiodiplodia pontae* | CMM 1277 | KT151794 | KT151791 | KT151797 |
| *Lasiodiplodia pontae* | CBS:117454 | MT587432 | MT592144 | MT592626 |
| *Lasiodiplodia pseudotheobromae* | CSF5802 | MT028604 | MT028770 | MT028936 |
| *Lasiodiplodia pseudotheobromae* | CGMCC 3.18068 | KX499902 | KX499940 | KX500015 |
| *Lasiodiplodia pseudotheobromae* | CSF6050 | MT028603 | MT028769 | MT028935 |
| *Lasiodiplodia pseudotheobromae* | HKAS 122658 | OL989258 | ON468648 | ON468654 |
| ***Lasiodiplodia pseudotheobromae*** | **CBS 116459 T** | **EF622077** | **EF622057** | **EU673111** |
| *Lasiodiplodia pyriformis* | CBS 121771 | EU101308 | EU101353 | KU887528 |
| ***Lasiodiplodia pyriformis*** | **CBS 121770 T** | **EU101307** | **EU101352** | **KU887527** |
| *Lasiodiplodia rubropurpurea* | WAC12536 | DQ103554 | DQ103572 | KP872425 |
| ***Lasiodiplodia rubropurpurea*** | **WAC12535 T** | **DQ103553** | **DQ103571** | **EU673136** |
| *Lasiodiplodia* sp.1 | CBS 125266 | MT587436 | MT592148 | KP872409 |
| *Lasiodiplodia* sp.2 | GuoLD01859 | KY783469 | KY848608 | KY848550 |
| *Lasiodiplodia sterculiae* | CBS:127106 | MN555400 | MT592146 | MT592631 |
| ***Lasiodiplodia sterculiae*** | **CBS 342.78 T** | **KX464140** | **KX464634** | **KX464908** |
| *Lasiodiplodia subglobosa* | CMM 4046 | KF234560 | KF226723 | KF254944 |
| ***Lasiodiplodia subglobosa*** | **CMM 3872 T** | **KF234558** | **KF226721** | **KF254942** |
| ***Lasiodiplodia swieteniae*** | **MFLUCC 18-0244 T** | **MK347789** | **MK340870** | **MK412877** |
| *Lasiodiplodia syzygii* | MFLUCC 19-0257 | MT990531 | MW016943 | MW014331 |
| *Lasiodiplodia syzygii* | GUCC 9719.2 | MW081991 | MW087101 | MW087104 |
| *Lasiodiplodia syzygii* | GUCC 9719.4 | MW081993 | MW087103 | MW087106 |
| ***Lasiodiplodia tenuiconidia*** | **CGMCC3.18061 T** | **KX499889** | **KX499927** | **KX500002** |
| *Lasiodiplodia thailandica* | CBS 138653 | KM006433 | KM006464 | / |
| *Lasiodiplodia thailandica* | CBS 138760 | KJ193637 | KJ193681 | / |
| *Lasiodiplodia thailandica* | DZP160119-9 | KY676788 | KY676797 | KY676794 |

| | | | | |
|---|---|---|---|---|
| *Lasiodiplodia thailandica* | DZP160123-13 | KY676789 | KY676798 | KY676795 |
| *Lasiodiplodia theobromae* | CBS 111530 | EF622074 | EF622054 | KU887531 |
| *Lasiodiplodia theobromae* | HKAS 122660 | OM030349 | ON468652 | ON468661 |
| *Lasiodiplodia theobromae* | HKAS 122659 | OM030345 | ON468653 | ON468662 |
| *Lasiodiplodia theobromae* | CBS:339.90 | KX464147 | KX464641 | KX464916 |
| ***Lasiodiplodia theobromae*** | **CBS 164.96 T** | **AY640255** | **AY640258** | **KU887532** |
| ***Lasiodiplodia tropica*** | **CGMCC 3.18477 T** | **KY783454** | **KY848616** | **KY848540** |
| *Lasiodiplodia vaccinii* | CGMCC 3.19023 | MH330319 | MH330329 | MH330326 |
| *Lasiodiplodia vaccinii* | CGMCC3.19256 | MK157139 | MK157166 | MK157157 |
| *Lasiodiplodia vaccinii* | CGMCC3.19255 | MK157138 | MK157165 | MK157156 |
| *Lasiodiplodia venezuelensis* | WAC12540 | DQ103548 | DQ103569 | KP872427 |
| ***Lasiodiplodia venezuelensis*** | **WAC 12539 T** | **DQ103547** | **DQ103568** | **KU887533** |
| *Lasiodiplodia viticola* | UCD2604MO | HQ288228 | HQ288270 | HQ288307 |
| ***Lasiodiplodia viticola*** | **UCD 2553AR T** | **HQ288227** | **HQ288269** | **HQ288306** |
| *Lasiodiplodia vitis* | CBS 124060 | KX464148 | KX464642 | KX464917 |

**Table S6. The names, isolate numbers, and corresponding GenBank accession numbers of the taxa used in Figure 12. The taxa produced in this study are indicated in red, and the type strains are indicated in bold with "T".**

| **Species name** | **Isolate No.** | **GenBank Accession No.** | | |
|---|---|---|---|---|
| | | **ITS** | **LSU** | ***tef1-a*** |
| *Aplosporella africana* | CBS 121777 | EU101315 | EU101380 | EU101360 |
| *Aplosporella africana* | CBS 121779 | EU101317 | EU101382 | EU101362 |
| *Aplosporella artocarpi* | KUMCC 21-0460 | OL989220 | OL989222 | ON468651 |

| | | | | |
|---|---|---|---|---|
| ***Aplosporella artocarpi*** | **B0391 T** | **KM006450** | **/** | **KM006481** |
| ***Aplosporella chromolaenae*** | **MFLUCC 17-1517 T** | **MT214340** | **NG_070506** | **/** |
| *Aplosporella ginkgonis* | CFCC 52442 | MH133916 | MH133933 | MH133950 |
| *Aplosporella ginkgonis* | CFCC 52443 | MH133917 | MH133934 | MH133951 |
| *Aplosporella ginkgonis* | CFCC 52444 | MH133918 | MH133935 | MH133952 |
| ***Aplosporella ginkgonis*** | **CFCC 89661 T** | **KM030583** | **KM030590** | **KM030597** |
| *Aplosporella ginkgonis* | CFCC 89660 | KM030582 | KM030589 | KM030596 |
| *Aplosporella hesperidica* | CBS 208.37 | MH867398 | / | KX464552 |
| *Aplosporella hesperidica* | CBS:732.79 | KX464083 | KX464239 | KX464553 |
| ***Aplosporella hesperidica*** | **MFLUCC:17-1518 T** | **MT214341** | **MT214435** | **/** |
| *Aplosporella javeedii* | CFCC 50054 | KP208840 | KP208843 | KP208846 |
| *Aplosporella javeedii* | CFCC 50053 | KP208839 | KP208842 | KP208845 |
| *Aplosporella javeedii* | CFCC 50052 | KP208838 | KP208841 | KP208844 |
| ***Aplosporella javeedii*** | **CFCC 89657 T** | **KM030579** | **KM030586** | **KM030593** |
| *Aplosporella macropycnidia* | CGMCC3.17726 | KT343649 | / | KX011177 |
| *Aplosporella macropycnidia* | CGMCC3.17727 | KT343647 | / | KX011175 |
| ***Aplosporella macropycnidia*** | **CGMCC3.17725 T** | **KT343648** | **/** | **KX011176** |
| *Aplosporella papillata* | CBS 121781 | EU101329 | EU101384 | EU101374 |
| *Aplosporella papillata* | CBS 121782 | EU101330 | EU101385 | EU101375 |
| ***Aplosporella papillata*** | **CBS 121780 T** | **EU101328** | **NG_070359** | **EU101373** |
| *Aplosporella prunicola* | STE-U 6326 | EF564375 | EF564377 | / |
| *Aplosporella prunicola* | STE-U 6327 | EF564376 | EF564378 | / |
| ***Aplosporella prunicola*** | **CBS 121167 T** | **KF766147** | **/** | **/** |
| ***Aplosporella thailandica*** | **MFLU 16-0615 T** | **KX423536** | **/** | **KX423537** |
| *Aplosporella yalgorensis* | MUCC 511 | EF591926 | EF591943 | EF591977 |
| ***Aplosporella yalgorensis*** | **MUCC 512 T** | **EF591927** | **EF591944** | **EF591978** |

| | | | | |
|---|---|---|---|---|
| ***Saccharata proteae*** | **CBS 115206 T** | **KF766226** | **GU357753** | **GU349030** |

**Table S7. The names, isolate numbers, and corresponding GenBank accession numbers of the taxa used in Figure 16. The taxa produced in this study are indicated in red, and the type strains are indicated in bold with "T".**

| **Species name** | **Isolate No.** | **GenBank Accession No** | | | |
|---|---|---|---|---|---|
| | | **ITS** | **LSU** | **SSU** | ***tub2*** |
| *Jattaea algeriensis* | STE-U 6201 | EU367446 | EU367456 | EU367462 | EU367466 |
| ***Pleurostoma hongkongense*** | **HKU 44 T** | **MT153150** | **NG_073830** | **MT158344** | **MT501300** |
| ***Pleurostoma ochraceum*** | **CBS 131321 T** | **MT153175** | **MT158397** | **MT158370** | **JX073271** |
| *Pleurostoma ootheca* | HKAS 122679 | OM017217 | OM017219 | OM017218 | ON468660 |
| *Pleurostoma ootheca* | CBS 115329 | MH862984 | MH874544 | MT158369 | MT501325 |
| ***Pleurostoma repens*** | **CBS 294.39 T** | **NR_135925** | **MT158398** | **NG_062675** | **MT501327** |
| *Pleurostoma richardsiae* | UAMH 8654 | MT153167 | MT158388 | MT158361 | MT501317 |
| *Pleurostoma richardsiae* | UAMH 8335 | MT153166 | MT158387 | MT158360 | MT501316 |
| *Pleurostoma richardsiae* | UAMH 5056 | MT153165 | MT158386 | MT158359 | MT501315 |
| *Pleurostoma richardsiae* | UAMH 5058 | MT153164 | MT158385 | MT158358 | MT501314 |
| *Pleurostoma richardsiae* | UAMH 5052 | MT153163 | MT158384 | MT158357 | MT501313 |
| *Pleurostoma richardsiae* | UAMH 4377 | MT153162 | MT158383 | MT158356 | MT501312 |
| *Pleurostoma richardsiae* | UAMH 10452 | MT153161 | MT158382 | MT158355 | MT501311 |
| *Pleurostoma richardsiae* | UAMH 10082 | MT153160 | MT158381 | MT158354 | MT501310 |
| *Pleurostoma richardsiae* | NCPF 2752 | MT153172 | MT158393 | MT158366 | MT501322 |
| *Pleurostoma richardsiae* | NCPF 7565 | MT153171 | MT158392 | MT158365 | MT501321 |

| | | | | | |
|---|---|---|---|---|---|
| *Pleurostoma richardsiae* | NCPF 2707 | MT153170 | MT158391 | MT158364 | MT501320 |
| *Pleurostoma richardsiae* | NCPF 2961 | MT153169 | MT158390 | MT158363 | MT501319 |
| *Pleurostoma richardsiae* | NCPF 2765 | MT153168 | MT158389 | MT158362 | MT501318 |
| *Pleurostoma richardsiae* | IFM 63541 | MT153173 | MT158395 | MT158368 | MT501324 |
| *Pleurostoma richardsiae* | IFM 63001 | MT153159 | MT158380 | MT158353 | MT501309 |
| *Pleurostoma richardsiae* | IFM 4926 | MT153158 | MT158379 | MT158352 | MT501308 |
| *Pleurostoma richardsiae* | IFM 41579 | MT153157 | MT158378 | MT158351 | MT501307 |
| ***Pleurostoma richardsiae*** | **CBS 270.33 T** | **MT153151** | **MH866889** | **NG_062674** | **MT501301** |
| *Pleurostoma richardsiae* | CBS 483.80 | MT153155 | MT158377 | MT158349 | MT501305 |
| *Pleurostoma richardsiae* | CBS 506.90 | MT153156 | MT505716 | MT505714 | MT501306 |
| *Pleurostoma richardsiae* | CBS 406.93 | MT153154 | MT158376 | MT158348 | MT501304 |
| *Pleurostoma richardsiae* | CBS 271.66 | NR_135933 | MT158394 | MT158367 | MT501323 |

## Supplementary information S1

The 97 strains are included in the combined gene analysis with 1157total character including gaps (ITS: 1–678 bp, β-tubulin: 679–1157 bp). Tree topology of the ML analysis was similar to the BI. The matrix had distinct alignment patterns, with the final ML optimization likelihood value of -15128.940737 (ln). All free model parameters were estimated by the RAxML model, with 854 distinct alignment patterns and 34.25% of undetermined characteristics or gaps. Estimated base frequencies were as follows: A = 0.225249, C = 0.265998, G = 0.234520, T = 0.274233, with substitution rates AC = 1.081904, AG = 3.209457, AT = 1.396424, CG = 0.972402, CT = 4.022213, GT = 1.000000. The gamma distribution shape parameter alpha = 0.626180 and the Tree-Length = 6.028220. The final average standard deviation of split frequencies at the end of total MCMC generations was calculated as 0.009873 in BI analysis. (Figure 1).

## Supplementary information S2

The 114 strains are included in the combined gene analysis with 4343 total characters including gaps (LSU: 1–849 bp, β-tubulin: 850–2681 bp, ITS: 2682–3269 bp, *rpb2*: 3270–4343 bp). Tree topology of the ML analysis was similar to the BI. The matrix had distinct alignment patterns, with the final ML optimization likelihood value of -89990.012031 (ln). All free model parameters were estimated by RAxML model, with 2653 distinct alignment patterns and 32.57% of undetermined characters or gaps. Estimated base frequencies were as follows: A = 0.239087, C = 0.268334, G = 0.251684, T = 0.240895, with substitution rates AC = 1.249855, AG = 4.147667, AT = 1.421319, CG = 0.936661, CT = 6.234325, GT = 1.000000. The gamma distribution shape parameter alpha = 0.899968 and the Tree-Length = 11.773179. The final average standard deviation of split frequencies at the end of total MCMC generations was calculated as 0.0098765 in BI analysis (Figure 3).

## Supplementary information S3

The 68 strains are included in the combined gene analysis with 2163 total characters including gaps (ITS: 1–574 bp, *tef1-α*: 575–934 bp, β-tubulin: 935–1677 bp, CAL: 1678–2163 bp). The tree topology of the ML analysis was similar to the BI. The matrix had distinct alignment patterns, with the final ML optimization likelihood value of -18655.070841 (ln). All free model parameters were estimated by RAxML model, with 1207 distinct alignment patterns and 22.01% of undetermined characters or gaps. Estimated base frequencies were as follows: A = 0.222883, C = 0.315096, G = 0.234336, T = 0.227685, with substitution rates AC = 1.504522, AG = 3.896321, AT = 1.359487, CG = 1.091388, CT = 5.495981, GT = 1.000000. The gamma distribution shape parameter alpha = 0.618776 and the Tree-Length = 3.289590. The final average standard deviation of split frequencies at the end of total MCMC generations was calculated as 0.009975 in BI analysis (Figure 5).

## Supplementary information S4

The 43 strains are included in the combined gene analysis with 3239 total characters including gaps (LSU: 1–899 bp, SSU: 900–1869 bp, ITS: 1870–2513 bp, rpb1: 2514–3239 bp). The tree topology of the ML analysis was similar to the BI. The matrix had distinct alignment patterns, with the final ML optimization likelihood value of -14760.740041 (ln). All free model parameters were estimated by the RAxML model, with 874 distinct alignment patterns and 36% of undetermined characters or gaps. Estimated base frequencies were as follows: A = 0.248563, C = 0.239637, G = 0.277107, T = 0.234693, with substitution rates AC = 2.518026, AG = 3.753497, AT = 3.014914, CG = 1.974550, CT = 10.451143, GT = 1.000000. The gamma distribution shape parameter alpha = 0.142975 and the Tree-Length = 2.542539 The final average standard deviation of split frequencies at the end of total MCMC generations was calculated as 0.009941 in BI analysis (Figure 7).

## Supplementary information S5

The 129 strains are included in the combined gene analyses with 1280 total characters including gaps (ITS: 1–524 bp, β-tubulin: 525–948 bp, *tef1-α*: 949–1280). The tree topology of the ML analysis was similar to the BI. The matrix had distinct alignment patterns, with the final ML optimization likelihood value of -6076.750023 (ln). All free model parameters were estimated by the RAxML model, with 459 distinct alignment patterns and 15.08% of undetermined characters or gaps. Estimated base frequencies were as follows: A = 0.206462, C = 0.307393, G = 0.255825, T = 0.230320, with substitution rates AC = 0.884829, AG = 3.200275, AT = 1.096972, CG = 1.056166, CT = 4.178329, GT = 1.000000. The gamma distribution shape parameter alpha = 0.658513 and the Tree-Length = 0.992396. The final average standard deviation of split frequencies at the end of total MCMC generations was calculated as 0.009654 in BI analysis (Figure 9).

## Supplementary information S6

The 30 strains are included in the combined gene analyses with 1501 total characters including gaps (LSU: 1–857 bp, ITS: 858–1162 bp, *tef1-α*: 1163–1501 bp). The tree topology of the ML analysis was similar to the BI. The matrix had distinct alignment patterns, with the final ML optimization likelihood value of -5432.091532 (ln). All free model parameters were estimated by the RAxML model, with 361 distinct alignment patterns and 24.98% of undetermined characters or gaps. Estimated base frequencies were as follows: A =0.222215, C = 0.258363, G = 0.272308, T = 0.247114, with substitution rates AC = 1.739870, AG = 4.289736, AT = 1.814791, CG = 2.163902, CT = 6.443658, GT = 1.000000. The gamma distribution shape parameter alpha = 0.462895 and the Tree-Length = 2.612554 The final average standard deviation of split frequencies at the end of total MCMC generations was calculated as 0.009881 in BI analysis (Figure 12).

## Supplementary information S7

The 28 strains are included in the combined gene analysis with 2648 total characters including gaps (LSU: 1–565 bp, SSU: 566–1593 bp, ITS: 1594–2175 bp, β-tubulin: 2176–2648 bp). The tree topology of the ML analysis was similar to the BI. The matrix had distinct alignment patterns, with the final ML optimization likelihood value of -7526.761364 (ln). All free model parameters were estimated by the RAxML model, with 412 distinct alignment patterns and 5.23% of undetermined characters or gaps. Estimated base frequencies were as follows: A = 0.236380, C = 0.264724, G = 0.279019, T = 0.219877, with substitution rates AC = 1.753295, AG = 2.247977, AT = 1.304010, CG = 1.106293, CT = 6.505394, GT = 1.000000. The gamma distribution shape parameter alpha = 0.443058 and the Tree-Length = 0.653975. The final average standard deviation of split frequencies at the end of total MCMC generations was calculated as 0.009836 in BI analysis (Figure 16).
